# Boosting Oxygen Electrocatalytic Activity of Fe–N–C Catalysts by Phosphorus Incorporation

**DOI:** 10.1021/jacs.2c12933

**Published:** 2023-02-06

**Authors:** Yazhou Zhou, Ruihu Lu, Xiafang Tao, Zijie Qiu, Guangbo Chen, Juan Yang, Yan Zhao, Xinliang Feng, Klaus Müllen

**Affiliations:** † Max Planck Institute for Polymer Research, Mainz 55128, Germany; ‡ School of Materials Science and Engineering, Jiangsu University, Zhenjiang 212013, Jiangsu, China; § State Key Laboratory of Silicate Materials for Architectures, International School of Materials Science and Engineering, Wuhan University of Technology, Wuhan 430070, Hubei, China; ∥ Center for Advancing Electronics Dresden (Cfaed) and Faculty of Chemistry and Food Chemistry, Technische Universität Dresden, Dresden 01062, Germany; ⊥ Max Planck Institute of Microstructure Physics, Weinberg 2, Halle (Saale) D-06120, Germany; # School of Science and Engineering, Shenzhen Institute of Aggregate Science and Technology, The Chinese University of Hong Kong, Shenzhen 518172, Guangdong, China

## Abstract

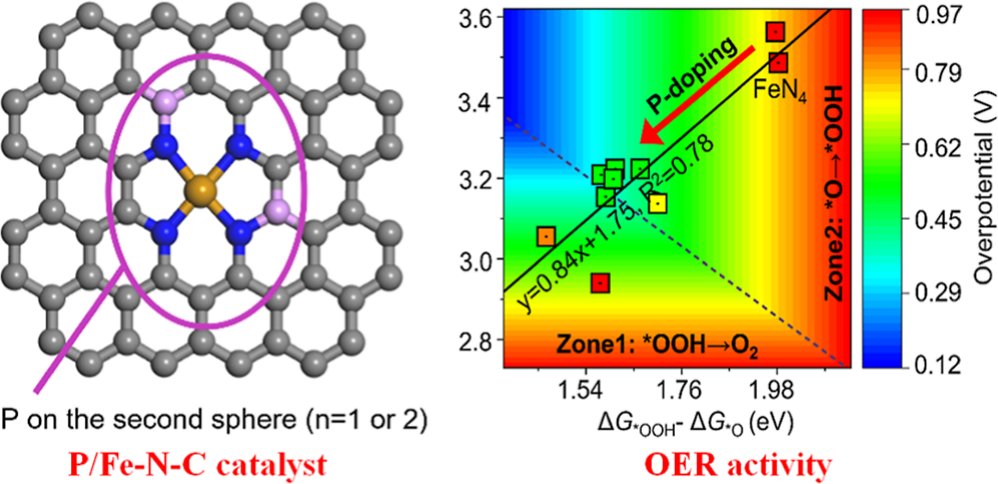

Nitrogen-doped graphitic carbon materials hosting single-atom iron (Fe–N–C) are major non-precious metal catalysts for the oxygen reduction reaction (ORR). The nitrogen-coordinated Fe sites are described as the first coordination sphere. As opposed to the good performance in ORR, that in the oxygen evolution reaction (OER) is extremely poor due to the sluggish O–O coupling process, thus hampering the practical applications of rechargeable zinc (Zn)–air batteries. Herein, we succeed in boosting the OER activity of Fe–N–C by additionally incorporating phosphorus atoms into the second coordination sphere, here denoted as P/Fe–N–C. The resulting material exhibits excellent OER activity in 0.1 M KOH with an overpotential as low as 304 mV at a current density of 10 mA cm^–2^. Even more importantly, they exhibit a remarkably small ORR/OER potential gap of 0.63 V. Theoretical calculations using first-principles density functional theory suggest that the phosphorus enhances the electrocatalytic activity by balancing the *OOH/*O adsorption at the FeN_4_ sites. When used as an air cathode in a rechargeable Zn–air battery, P/Fe–N–C delivers a charge–discharge performance with a high peak power density of 269 mW cm^–2^, highlighting its role as the state-of-the-art bifunctional oxygen electrocatalyst.

## Introduction

Rechargeable zinc (Zn)–air batteries have emerged as attractive components of energy technology owing to their high energy density and environmental friendliness together with the abundance of Zn resources.^1,2^ The energy efficiency of the Zn–air battery is primarily determined by the oxygen electrocatalysts of the air electrode, where the oxygen reduction reaction (ORR) and oxygen evolution reaction (OER) take place as alternating steps for discharging and charging processes, respectively.^3^ However, because of their sluggish kinetics of multi-step four-electron transfer during the electrochemical process, Zn–air batteries are still hampered by very large potential gaps (>0.85 V) and low energy efficiencies.^4^ To date, precious group metals (PGMs) such as platinum (Pt) and iridium (Ir) still serve as benchmark catalysts for ORR and OER,^5,6^ respectively, whose high costs exclude widespread application.^7,8^

In the past decade, single-atom transition metal and nitrogen co-doped graphitic carbon (TM–N-C) materials have been developed as PGM-free catalysts with excellent ORR activity and stability in alkaline media.^9,10^ The catalytically active site consists of a single transition metal center (i.e., TM) and N ligands, giving rise to TMN_*x*_C_*y*_ units immobilized in N-doped graphitic carbon supports.^11−14^ The N and C atoms are located at the first and second coordination sphere of the transition metal center, respectively^15^ (Figure S1). Among PGM-free catalysts, Fe–N–C materials are the most promising catalysts for ORR.^16^ FeN_4_ is the most active entity whereby the Fe center adsorbs oxygen molecules and catalyzes the subsequent four-electron transfer ORR (O_2_ → OOH* → O* → OH* → OH^–^).^17−20^ Although the newly reported Fe–N–C materials displayed a superior ORR performance than that of commercial Pt/C catalysts in alkaline solution by improving the density and accessibility of FeN_4_ units and enhancing the intrinsic activity of FeN_4_ for ORR by tailoring the local carbon structure (e.g., modulation by another light heteroatom),^21^ their OER performance was still extremely poor.^22,23^ Achieving both high ORR and OER performance on Fe–N–C is challenging but is critically important for the practical applications of these catalysts in rechargeable Zn–air batteries. The electrocatalytic activity is significantly affected by the adsorption behavior of the abovementioned oxygen-containing intermediates on the active center.^24^ The reverse process of ORR, the OER on Fe–N–C, is kinetically sluggish mainly due to the slow O–O coupling process because of the strong O* binding strength on central Fe.^25^ As a result, the catalytic conversion of *O into *OOH is the rate-determining step. Therefore, weakening the O* adsorption on the Fe center is supposed to increase the OER kinetics. Current experimental research in this direction mainly focuses on the regulation of the first or second coordination sphere, including coordination numbers, the nature of ligands,^26^ and interactions between single metals.^27−29^ These strategies were successfully used to promote the ORR activity.^30^ Unfortunately, these strategies also weaken the OOH* adsorption, hampering the deprotonation of *OOH to release O_2_ limited to the scaling relation between OH* and OOH* intermediates (Δ*G*_OOH*_ = Δ*G*_OH*_ + 3.2 ± 0.2 eV), hampering the promotion of OER kinetics.^31^ Therefore, balancing the *OOH/*O adsorption by weakening the O* adsorption, while at the same time enhancing the *OOH adsorption, is key for improving the OER kinetic activity on FeN_4_.^32,33^

Toward that end, the Fe–N–C catalyst containing additional phosphorus incorporation was synthesized. This new material, named P/Fe–N–C, was obtained by the carbonization of the ferric phytate-modified Fe-doped zeolitic imidazolate framework (PA@Fe-ZIF-8). Spectroscopy characterization revealed that FeN_4_ moieties were embedded into graphitic carbon, while phosphorus was introduced into the second coordination sphere. P/Fe–N–C delivered a significantly improved OER activity with an overpotential of only 304 mV at a current density of 10 mA cm^–2^, as well as an excellent ORR performance in the 0.1 M KOH electrolyte. The OER activity is comparable to that of the IrO_2_ benchmark (296 mV) and outperforms that of phosphorus-free Fe–N–C (450 mV). The new P/Fe–N–C catalyst, thus, qualifies as a cutting-edge bifunctional oxygen electrocatalyst. When used in an air electrode, the resulting Zn–air battery exhibited a high maximum power density of 269 mW cm^–2^ together with outstanding charge–discharge efficiency and stability. Density functional theory (DFT) calculations suggest that the presence of phosphorus results in a local distortion around Fe centers and changes the behavior of*OOH/*O adsorption. Furthermore, a new negative linear relation for *OOH/*O intermediates is found, which breaks the conventional scaling relation and balances the *OOH/*O adsorption behavior.

## Results and Discussion

### Material Synthesis and Characterization

**Figure 1:**
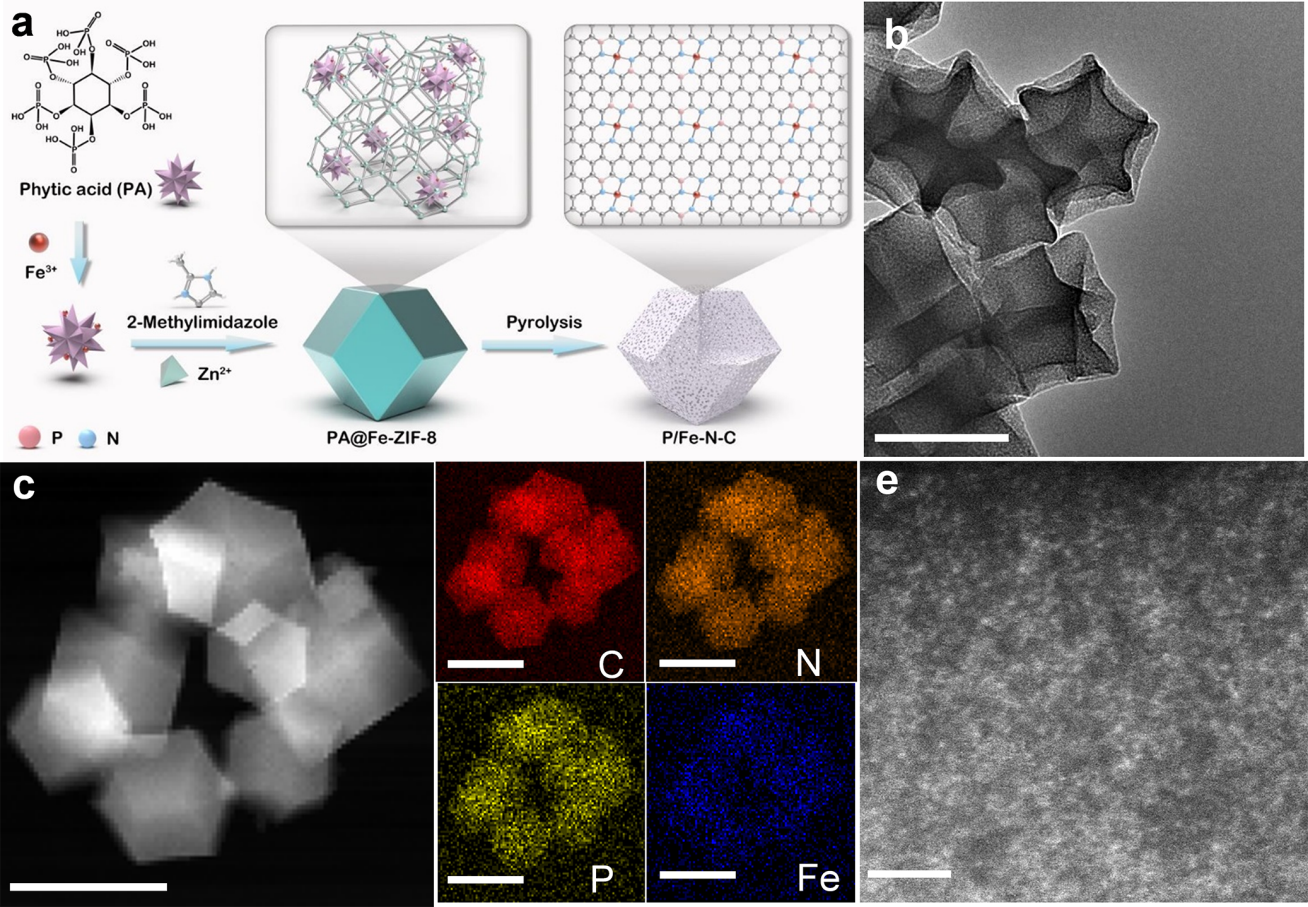
Synthesis and characterization of the P/Fe–N–C material. (a) Schematic illustration of the synthesis of P/Fe–N–C. (b) TEM image. (c) HAADF-STEM and (d) related elemental mapping images demonstrating the distribution of C (red), N (orange), P (yellow), and Fe (blue) elements. (e) Atomic-resolution HAADF-STEM image. Scale bar: (b–d) 150 and (e) 2 nm.

The synthesis of the P/Fe–N–C material is illustrated in Figure 1a. Briefly, Fe(NO_3_)_3_·6H_2_O and phytic acid were mixed in a methanol solution to form the ferric phytates at 60 °C. Afterward, methanol solutions of Zn(NO_3_)_2_·6H_2_O and 2-methylimidazole were added sequentially. The products, named PA@Fe-ZIF-8, were collected after reacting for 24 h. The transmission electron microscopy (TEM) image revealed dodecahedron-shaped PA@Fe-ZIF-8 nanoparticles (Figure S2). The X-ray diffraction (XRD) analysis of PA@Fe-ZIF-8 and Fe-ZIF-8 showed identical diffraction patterns (Figure S3), indicating that the introduction of ferric phytates did not disturb the crystallization of ZIF-8. PA/Fe-ZIF-8 was then thermally treated at 1000 °C for 1 h under an Ar atmosphere. Thereby, ZIF-8 transformed into nitrogen-doped porous graphitic carbon to host single Fe atoms. Ferric phytates can not only offer a high amount of stabilized Fe atoms to form highly dispersed FeN_4_ moieties but also provide phosphorus sources for the formation of the second coordination sphere around the Fe centers. The reference samples, denoted as P/Fe@N–C with Fe-based nanoparticles (Figure S4) and phosphorus-free Fe–N–C, were synthesized by identical protocols with a high loading of PA and Fe ions and without the utilization of PA, respectively (Figure S5). The detailed protocol is given in the Methods section.

**Figure 2:**
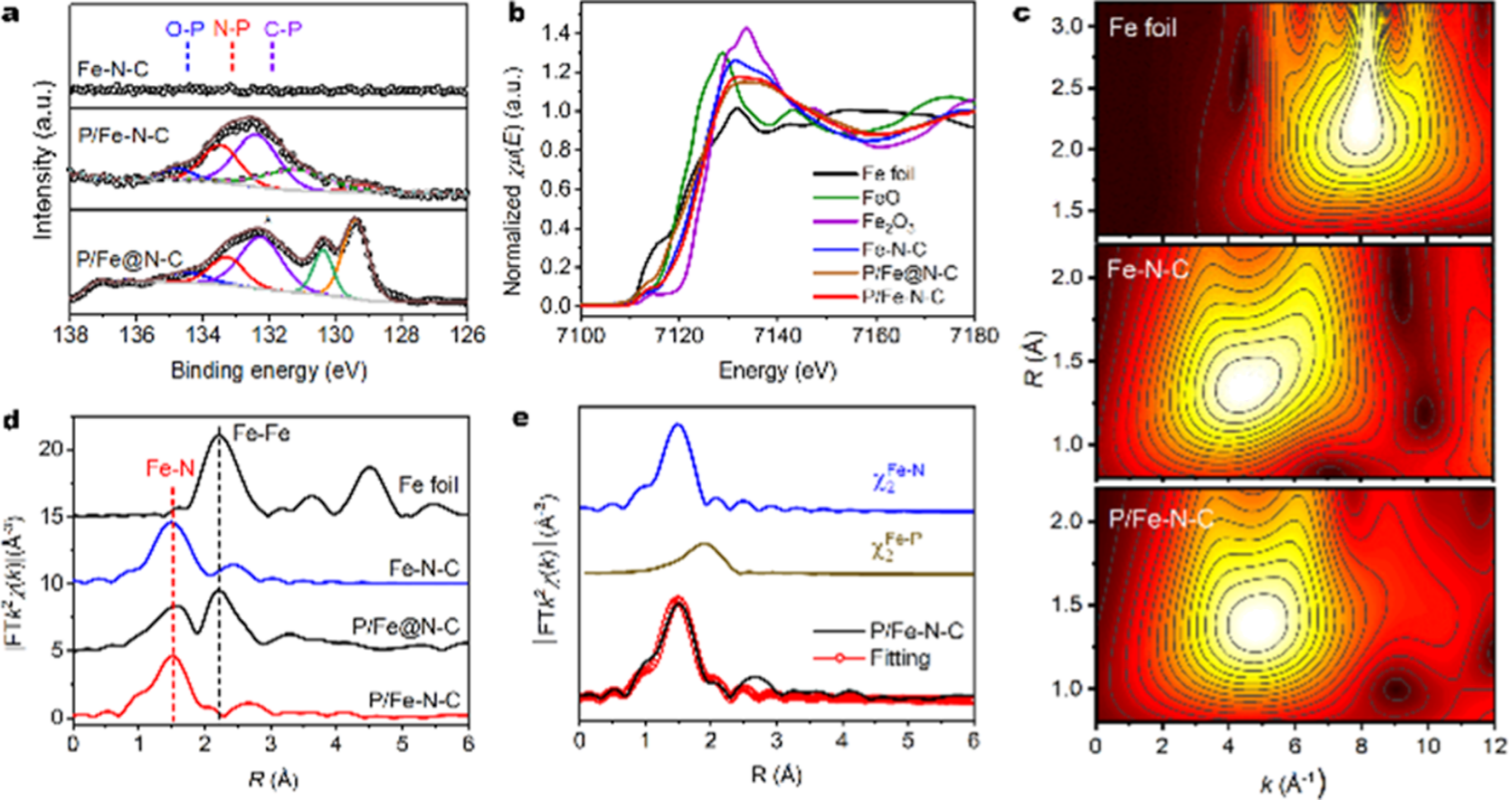
Structural characterization. (a) High-resolution P 2p XPS spectra of Fe–N–C, P/Fe–N–C, and P/Fe@N–C. (b) Fe K-edge XANES spectra of Fe–N–C, P/Fe–N–C, P/Fe@NC, Fe_2_O_3_, FeO, and Fe foil references. (c) *k*^3^-weighted wavelet-transformed Fe K edge EXAFS spectra of Fe–N–C, P/Fe–N–C, and Fe foil. (d) *k*^3^-weighted Fourier-transformed EXAFS spectra of Fe–N–C, P/Fe–N–C, P/Fe@NC, and Fe foil. (e) EXAFS fitting curves of P/Fe–N–C.

The structure and morphology of the resulting Fe–N–C and P/Fe–N–C materials were first examined using scanning electron microscopy and TEM. As shown in Figures 1b and S5a, both P/Fe–N–C and Fe–N–C retained the original dodecahedron morphology of Fe-ZIF-8 particles. No Fe-containing nanoparticles (e.g., Fe and/or Fe_2_P) were detected in the TEM and high-angle annular dark-field scanning TEM (HAADF-STEM) images (Figures 1c and S5b,c). Corresponding elemental mapping images revealed the homogeneous distribution of C, N, P, and Fe elements for P/Fe–N–C and the absence of P in Fe–N–C (Figures 1d and S5d,e). Furthermore, the aberration-corrected HAADF-STEM images of P/Fe–N–C and Fe–N–C indicated that Fe was atomically dispersed within the carbon support (Figures 2e and S5f). The XRD patterns of the two samples displayed two broad peaks at 24.3 and 43.7°, corresponding to the (002) and (101) planes of graphitic carbon, respectively (Figure S6). No diffraction peaks related to crystalline Fe species (e.g., Fe, Fe_3_C, or Fe_3_N nanoparticles) were observed. The Raman spectra of Fe–N–C and P/Fe–N–C exhibited two peaks at 1350 and 1590 cm^–1^, corresponding to the D band (disordered sp^3^ carbon) and G band (graphitic sp^2^ carbon) of graphitic carbon, respectively (Figure S7). The Brunauer–Emmett–Teller surface area and the total pore volume of P/Fe–N–C were measured to be 950 m^2^ g^–1^ and 1.2 cm^3^ g^–1^, respectively, that is, higher than those of Fe–N–C at 684 m^2^ g^–1^ and 1.0 cm^3^ g^–1^, respectively (Figure S8). This could be due to gas released from the decomposition of ferric phytates, which enhanced porosity. The Fe content in P/Fe–N–C was 1.70 wt % according to inductively coupled plasma-optical emission spectroscopy and thus slightly higher than the value of 1.53 wt % for Fe–N–C.

X-ray photoelectron spectroscopy (XPS) was utilized to probe the elemental information of Fe–N–C and P/Fe–N–C (Tables S1 and S2). As depicted in Figure S9, the XPS survey spectra confirm the existence of C, N, and Fe in both materials. In contrast to Fe–N–C, a new peak located at ∼133 eV was observed, which was assigned to P 2p. The high-resolution P 2p XPS spectrum of P/Fe–N–C displays three peaks at 132.1, 133.2, and 134.3 eV (Figure 2a), which can be assigned to C–P, P–N, and P–O groups, respectively.^34^ The results suggest the successful incorporation of phosphorus atoms into the graphitic carbons. The high-resolution O 1s spectrum 1s reveals the absence of the Fe–O species in the P/Fe–N–C material (Figure S10). Synchrotron-radiation-based X-ray absorption near-edge structure (XANES) and extended X-ray absorption fine structure (EXAFS) measurements were then carried out to investigate the electronic structure and coordination modes of the P/Fe–N–C, P/Fe@NC, and Fe–N–C samples. Fe K-edge XANES was first applied to analyze the oxidation state of the Fe atoms in each sample by comparing the edge position of the samples with that of standard Fe foil, FeO, and Fe_2_O_3_. As revealed in Figure 2b, the absorption edges of P/Fe–N–C and Fe–N–C are located in between FeO and Fe_2_O_3_, implying that the valence of Fe species in two samples is between 2+ and 3+.^35^ Compared to Fe–N–C, P/Fe–N–C displays a positive absorption edge, suggesting a higher valence state of Fe. The presence of phosphorus atoms can thus modulate the electronic structure of the central single Fe atom of Fe–N–C materials. Fe K-edge EXAFS was applied to examine the local coordination geometry of Fe sites in P/Fe–N–C and Fe–N–C. The wavelet-transformed EXAFS plots of Fe–N–C, P/Fe–N–C exhibit only one intensity maximum at ∼5.1 Å^–1^ in *k* space, similar to FePc, implying an analogous Fe–N first-shell coordination (Figure 2c).^36^ In the Fourier-transformed EXAFS *R*-space plot (Figure 2d), both P/Fe–N–C and Fe–N–C showed one main peak located at ∼1.52 Å, contributing to the Fe–N first coordination sphere. Compared with Fe foil and P/Fe@NC, the Fe–Fe coordination peak at ∼2.2 Å was absent in both P/Fe–N–C and Fe–N–C. This reveals the atomically dispersed nature of Fe sites.^36^ EXAFS fitting analysis was adopted to determine the coordination number and interatomic bonding distance of the central Fe in P/Fe–N–C and Fe–N–C. The best-fitting result for P/Fe–N–C was obtained by using two backscattering pathways of Fe–N and Fe–P (Figure 2e), and only the Fe–N pathway can be fitted for Fe–N–C. The coordination numbers of Fe–N and Fe–P for P/Fe–N–C were calculated as 4.1 ± 0.5 and 2.2 ± 0.7 at distances of 1.97 ± 0.02 Å and 2.32 ± 0.04 Å, respectively (Table S3). Considering the coordination numbers, we proposed a configuration of FeN_4_-P_*n*_ (*n* = 1 or 2) in which N is located in the first coordination sphere and P is located in the second coordination sphere of the Fe center. In contrast, the single-atom Fe in Fe–N–C is coordinated with four N atoms, the typical FeN_4_ moiety.

### OER and ORR Activities

**Figure 3:**
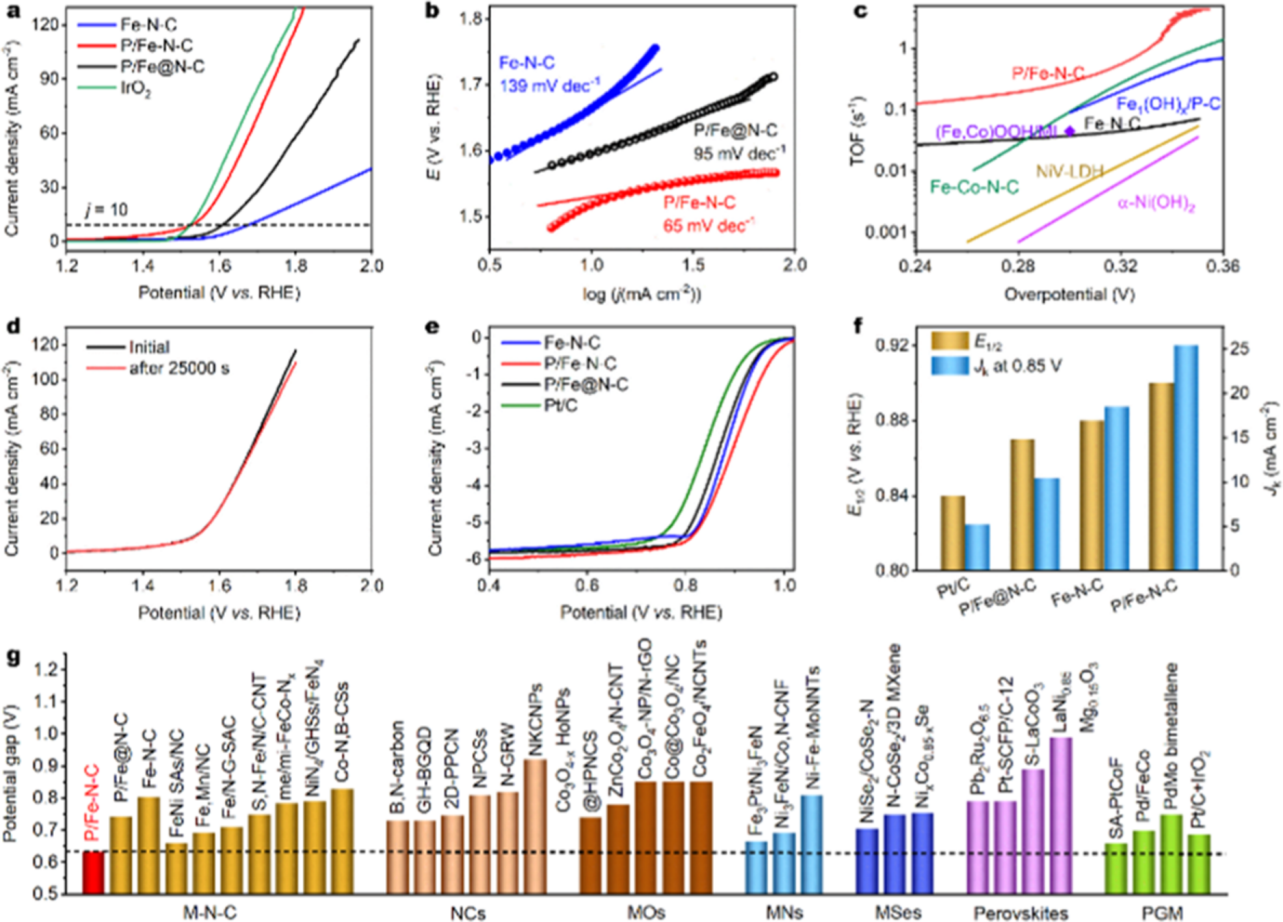
Electrocatalytic performance of P/Fe–N–C. (a) OER LSV curves of Fe–N–C, P/Fe–N–C, P/Fe@N–C, and IrO_2_/C electrocatalysts. (b) OER Tafel plots of Fe–N–C, P/Fe–N–C, and P/Fe@N–C. (c) TOF values for OER of the P/Fe–N–C catalyst and other recently reported OER catalysts based on earth-abundant metals, including (Fe,Co)OOH/MI (ref (49)), Fe_1_(OH)_*x*_/P–C(ref (50)), NiV-LDH (ref (51)), α-Ni(OH)_2_ (ref (52)), and Fe–Co–N–C (ref (53)). The TOF values are based on the total amounts of metal for all catalysts. (d) LSV curves of the P/Fe–N–C electrocatalyst before and after 25,000 s stability test. (e) ORR polarization curves and (f) *J*_k_ at 0.85 V and *E*_1/2_ for Fe–N–C, P/Fe–N–C, P/Fe@N–C, and Pt/C electrocatalysts. (g) Comparison of the potential gaps for the P/Fe–N–C and state-of-the-art bifunctional oxygen electrocatalysts. The details are shown in Table S6.

The electrocatalytic OER performance of P–Fe–N–C was investigated by linear sweep voltammetry (LSV) using a rotating disk electrode (RDE) technique in an O_2_-saturated 0.1 M KOH electrolyte solution. For comparison, a commercial IrO_2_ catalyst, Fe–N–C, and P/Fe@NC were evaluated under the same conditions. All potentials were referenced to the reversible hydrogen electrode. As depicted in Figure 3a, the Fe–N–C presented a high onset OER overpotential of ∼350 mV. The result is consistent with the reported values, in which single-atom Fe–N–C materials reveal very sluggish OER kinetics.^37^ In contrast, on P/Fe–N–C, oxygen generation occurred at an overpotential of only ∼130 mV, which was substantially lower than the value of ∼270 mV for the commercial IrO_2_ catalyst. Outstandingly, the current density of P/Fe–N–C reached 10 mA cm^–2^ (*j*_10_) at a substantially decreased overpotential of 304 mV (without *iR* compensation), which was comparable with that of IrO_2_ (296 mV) and much lower than those of P/Fe@NC (384 mV) and Fe–N–C (450 mV). In addition, the Tafel slope of P/Fe–N–C was determined as ∼65 mV decade^–1^, much lower than ∼88 mV decade^–1^ for the IrO_2_ (Figures 3b and S11). The electrochemical impedance spectroscopy of P/Fe–N–C plotted in Figure S12 further manifested a faster OER kinetic process than that of Fe–N–C. These results clearly indicate that the sluggish OER kinetics of Fe–N–C is significantly accelerated by the incorporation of phosphorus atoms. Remarkably, the OER performance of P/Fe–N–C is superior to that of previously reported single-atom M–N-C electrocatalysts such as Fe–N_*x*_–C (600 mV),^38^ S,N–Fe/N/C-CNT (370 mV),^39^ Ni-NHGF (331 mV),^40^ Ni–N_4_/GHSs/Fe–N_4_ (390 mV),^41^ Fe–Ni–N–P–C (337 mV),^42^ and Fe,Mn/N–C (390 mV)^43^ and even comparable or superior to those of the reported state-of-the-art metal oxides/nitrides/phosphides/selenide-based OER electrocatalysts (Table S4). Furthermore, the reaction mechanism was determined by the rotating ring-disk electrode (RRDE) technique. A very low ring current was detected, suggesting negligible hydrogen peroxide formation (Figure S13). This result means exclusive generation of O_2_ during the OER process through a four-electron process, that is, 4OH^–^ → O_2_ + 2H_2_O + 4e^–^. Moreover, the double-layer capacitance (*C*_dl_) value suggests a large electrochemical active surface area of P/Fe–N–C, which would contribute to the enhanced catalytic activity (Figure S14). To evaluate the intrinsic activity of P/Fe–N–C, we estimated the turnover frequency (TOF). At an overpotential of 300 mV, the TOF of P/Fe–N–C was calculated as 0.29 s^–1^, which is 7.6 times higher than the value for Fe–N–C (Figure 3c). Also, we compared the TOF of the P/Fe–N–C catalyst at different overpotentials with the TOF values of some recently reported non-precious-metal catalysts. Clearly, the comparison highlights P/Fe–N–C as being among the most active OER catalysts with the highest atom utilization efficiency. Catalytic durability is another vital criterion for evaluating the OER performance of an electrocatalyst which was determined by holding a constant current density (i.e., *j*_10_) for 25,000 s (Figure S15). As revealed in Figure 3d, the OER overpotential of the P/Fe–N–C at *j*_10_ increased by only 2 mV. Some loss of activity was observed after holding at a constant voltage of 1.53 V for 10 h. The structural and chemical compositional changes in the P/Fe–N–C after the durability tests were examined using Raman spectroscopy, XPS (Figure S16), and HRTEM analyses (Figure S17).

The electrocatalytic ORR tests of P/Fe–N–C were also conducted by the RDE technique in a 0.1 M KOH employing a commercial Pt/C catalyst (20% Pt, Fuelcellstore), Fe–N–C, and P/Fe@N–C as references. As shown in Figure 3e, Fe–N–C displayed excellent ORR performance with an onset potential of 0.99 V. The ORR activity of P/Fe–N–C was slightly improved with an onset potential of 1.01 V. Noticeably, a half-wave potential (*E*_1/2_) of 0.90 V was higher than those of 0.88 V for Fe–N–C, 0.87 V for P/Fe@N–C, and 0.84 V for Pt/C benchmark. In addition, the kinetic current density (*J*_k_) of the P/Fe–N–C reached 25.4 mA cm^–2^ at 0.85 V, which was 4.9 times higher than the value for Pt/C (Figure 3f). Using the RRDE method, the H_2_O_2_ yield of the P/Fe–N–C sample was determined as <1.5% in a potential range from 0.4 to 0.9 V. The corresponding electron-transfer number was larger than 3.9 (Figure S18), suggesting the desired four-electron ORR process. In addition, the P/Fe–N–C material presented good ORR stability (Figure S19). The site density for ORR was quantified by the in situ electrochemical method by means of nitrite absorption followed by reductive stripping. The corresponding TOF was then evaluated based on the stripping charge. The P/Fe–N–C displayed a higher site density of 21.8 μmol g^–1^ and a lower TOF of 2.0 s^–1^ at 0.85 V compared to those of Fe–N–C (13.9 μmol g^–1^ and 2.5 s^–1^) (Figure S20 and Table S5). These results indicate that the slightly enhanced ORR activity of P/Fe–N–C can mainly be attributed to the high intrinsic activity and increased density of active sites as a result of high Fe loading and surface area.

The bifunctional oxygen electrocatalytic performance of P/Fe–N–C was estimated by calculating the potential gap (Δ*E*) between the OER potential at 10 mA cm^–2^ (*E*_*j*=10_) and ORR *E*_1/2_. Remarkably, the P/Fe–N–C demonstrated a small Δ*E* of 0.63 V, and thus, it was substantially lower than the value of 0.69 V for the Pt/C–IrO_2_ couple and those for state-of-the-art bifunctional oxygen electrocatalysts including single-atom M-N-C, metal-free materials, metal oxides/nitrides/phosphides/selenides, perovskites, and PGM-based electrodes (Figure 3g and Table S6). Representative examples are 0.69 V for Fe,Mn/NC,^43^ 0.73 V for B-doped graphene quantum dots anchored on a graphene hydrogel (GH-BGQD),^44^ 0.74 V for Co_3_O_4–*x*_ HoNPs@HPNCS,^45^ 0.69 V for Ni_3_FeN/Co,N–CNF,^46^ 0.79 V for Pb_2_Ru_2_O_6.5_,^47^ and 0.75 V for PdMo bimetallene.^48^ These results implied that the P/Fe–N–C is one of the best candidates for bifunctional oxygen electrocatalysis.

### Rechargeable Zn–Air Battery Performance

**Figure 4:**
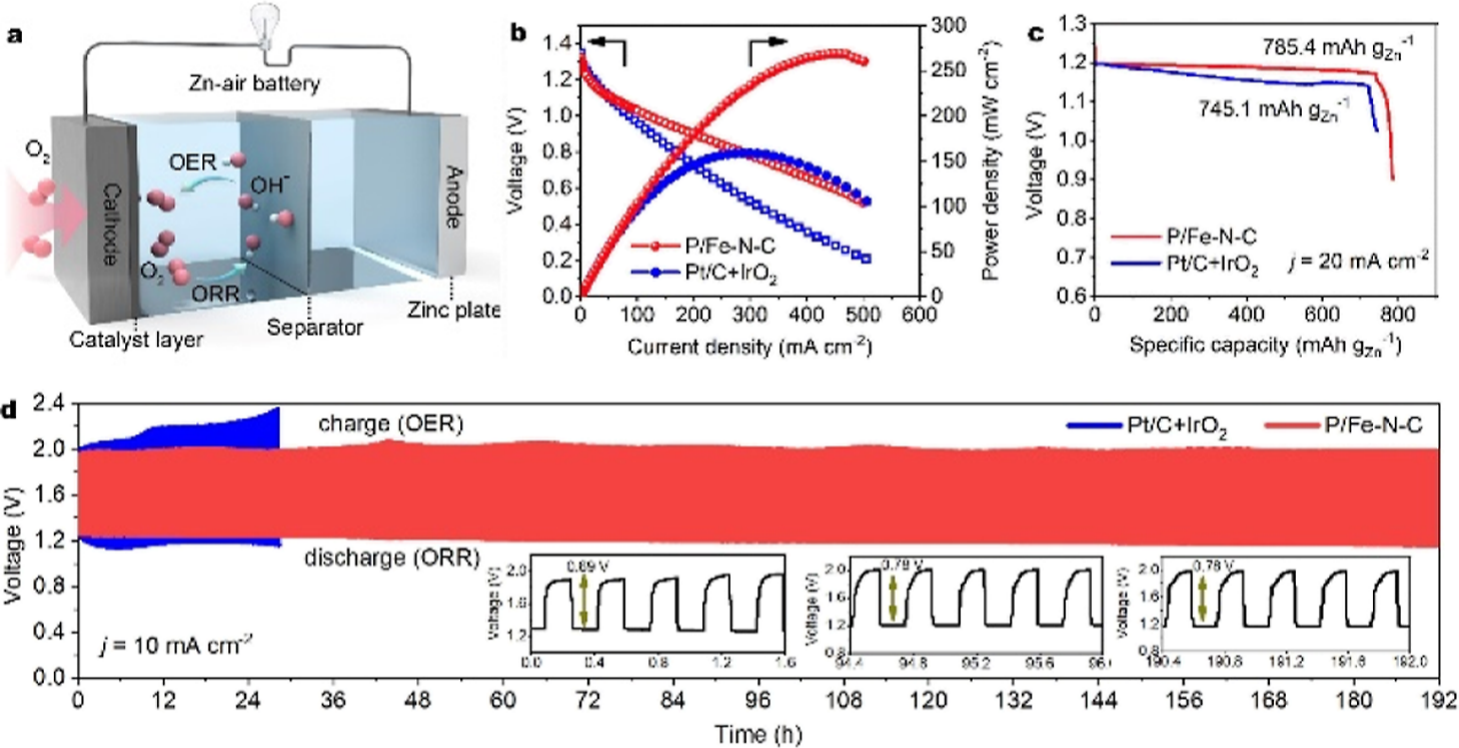
Rechargeable Zn–air battery performance. (a) Scheme showing the as-assembled rechargeable Zn–air battery. (b) Discharge polarization curves and corresponding power density plots of the rechargeable Zn–air batteries using the P/Fe–N–C and Pt/C–IrO_2_ couple as air electrodes, respectively. (c) Zn-mass-normalized specific capacities at a current density of 20 mA cm^–2^. (d) Discharge/charge cycling curves of Zn–air batteries using the P/Fe–N–C and Pt/C–IrO_2_ couple as air electrodes at 10 mA cm^–2^.

To evaluate its practical application in energy devices, a rechargeable Zn–air battery was assembled utilizing P/Fe–N–C as the oxygen catalyst of the air electrode in a 6.0 M KOH electrolyte containing 0.2 M Zn(OAc)_2_ (Figure 4a). A reference Zn–air battery was also constructed and tested by using a Pt/C + IrO_2_ coupled catalyst (with the same mass ratio) as the air electrode. As shown in Figure S17, the open-circuit voltage of the P/Fe–N–C reached 1.48 V. The corresponding maximum power density of the P/Fe–N–C-based battery was 269 mW cm^–2^ and higher than that of Pt/C + IrO_2_ (159 mW cm^–2^) (Figure 4b). It delivered a specific capacity of 785 mA h g_Zn_^–1^ at a discharge current density of 20 mA cm^–2^, corresponding to ∼96% utilization of the theoretical capacity (∼820 mA h g_Zn_^–1^)^39^ (Figure 4c). To investigate the cycling performance of the Zn–air batteries, the galvanostatic charge and discharge test was performed at 10 mA cm^–2^ with 20 min per cycle (10 min for discharge, 10 min for charge). After 96 h, the potential gap for charge and discharge only increased by 0.09 V. Then there was no noticeable degradation over 192 h, suggesting good electrocatalytic durability in the alternative OER and ORR processes (Figure 4d). In sharp contrast, the battery with the Pt/C + IrO_2_ catalyst lost its activity after 500 cycles.

### Theoretical Investigation of the Role of Phosphorus

**Figure 5:**
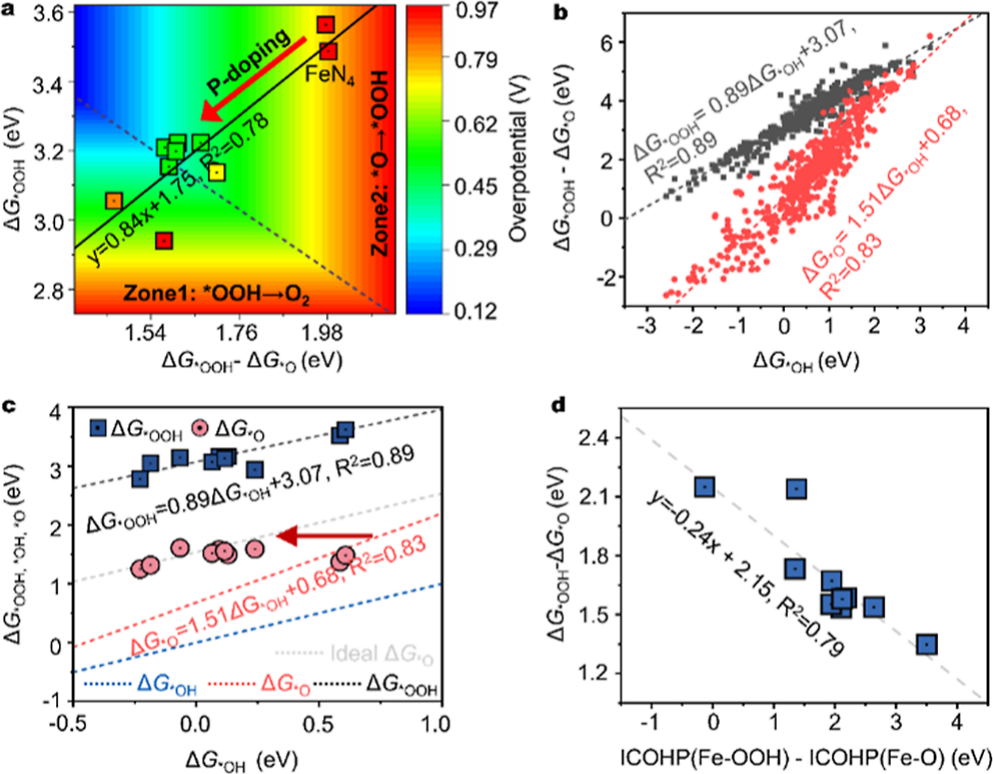
Fundamental understanding of the P effect on Fe active sites by using DFT calculations. (a) Contour plot of OER overpotential as a function of Gibbs adsorption energies [(ΔG_*OOH_ – ΔG_*O_) along the *x*-axis and ΔG_*OOH_ along the *y*-axis]. The inset is the scaling relation ΔG_*OOH_ = 0.84 (ΔG_*OOH_ – ΔG_*O_) + 1.75. (b) Scaling relations of adsorption energies among OER intermediates. The blue, red, and black dashed lines are obtained by fitting data from previous studies, and the gray dashed line is the middle line between ΔG_*OOOH_ and ΔG_*OH_, indicating the ideal line of ΔG_*O_. (c) Scaling relation of adsorption energies for different OER intermediates on the P/Fe–N–C. The dotted line denotes the statistics scaling relation on TM-N_4_C_12_ originated in figure b. (d) Linear relation between ICOHP(Fe–OOH) – ICOHP(Fe–O) and ΔG_*OOH_ – ΔG_*O_.

First-principles DFT calculations were conducted to shed light on the influence of phosphorus on the OER activity of P/Fe–N–C. We constructed 23 possible structures. Two kinds of models were considered, comprising one or two phosphorus atoms within the second coordination sphere of the Fe center (i.e., FeN_4_–P_1_ and FeN_4_–P_2_) (Figure S21). By screening the bond length and energy of formation for the Fe–P bond, nine models (Table S7) were fitted for our experimental analysis. A local distortion was found due to the larger atomic radius of the phosphorus atom with respect to carbon. This induces structural distortions depending upon the number and position of P-dopants together with lengthening the Fe–N bonds (Table S8) and generates tensile strain. Strain effects represent an efficient strategy to tune the local environment of active sites and thus the oxygen binding ability.^31,33^ The adsorption behavior of the OER intermediates (i.e., OH*, O*, and OOH*) on the catalyst models was examined (Table S9), and the corresponding Gibbs free energy profiles for the OER are displayed in Figure S22. It can be concluded from Figure 5a that the OER activity on all the catalyst models can be described as a function of the Gibbs adsorption energies (ΔG_*OOH_ – ΔG_*O_) and ΔG_*OOH_. The former corresponds to the steps of O–O coupling (*O → *OOH), and latter corresponds to O_2_ release (*OOH → O_2_). On pristine Fe–N–C, the OER overpotential is as large as 0.92 eV for O–O coupling. This explains the poor OER activity of the reported Fe–N–C materials.^40^ In contrast, in all P/Fe–N–C models, the OER activities are improved, benefitting from the increased ΔG_*OOH_ and decreased (ΔG_*OOH_ – ΔG_*O_) values. The effect of alternative structures in the P/Fe–N–C catalyst on OER activity was also investigated, including vacancy defects, N doping, P doping, N, P co-doping, dual-atom Fe, and P-doped Fe–N–C, which contain different distances between Fe and P atoms (Figures S23–S27 and Tables S10 and S11). One can conclude that the introduction of phosphorus into the second coordination sphere of the Fe center leads to an optimal OER performance by tuning the *OOH/*O adsorption. One can conclude that the introduction of phosphorus into the second coordination sphere of the Fe center leads to an optimal OER performance by tuning the *OOH/*O adsorption.

To further understand the mechanism of augmented OER activity, the change in *OOH/*O adsorption behavior was investigated. We collected approximately 500 pieces of data from reference papers to plot relatively precise scaling relations of TMN_4_-C_12_ configuration in Figure 5b and the dashed line in Figure 5c. They are ΔG_*OOH_ = 0.89ΔG_*OH_ + 3.07, *R*^2^ = 0.89 and ΔG_*O_ = 1.51ΔG_*OH_ + 0.68, *R*^2^ = 0.83, respectively. These scaling relations slightly differ from the reported one, that is, Δ*G*_OOH*_ = Δ*G*_OH*_ + 3.2 on metal oxide.^54^ Generally, an ideal OER catalyst should have an optimal ΔG_*O_ value between ΔG_*OH_ and ΔG_*OOH_. When plotting ΔG_*O_ versus ΔG_*OH_ (1.51) for the single-atom catalysts, ΔG_*O_ deviates more from the value between ΔG_*OH_ and ΔG_*OOH_. Therefore, these catalysts exhibit a large difference of (ΔG_*O_ – ΔG_*OOH_) and a high OER overpotential. This is in good agreement with the big change in free energy by converting *O into *OOH (Figure S22), indicating a sluggish O–O coupling process on Fe–N–C. The newly linear correlation between ΔG_*OOH_ – ΔG_*O_ and ΔG_*OOH_: ΔG_*OOH_ = 0.84 (ΔG_*OOH_ – ΔG_*O_) + 1.75, *R*^2^ = 0.78 on P/Fe–N–C systems (Figure 5a) implies that the ΔG_*OOH_ is negatively correlated with ΔG_*O_. This differs from predictions of earlier scaling relations.^33^ It can be seen from Figure 5c that the *OOH adsorption energies on P/Fe–N–C still fulfill the scaling relations of TMN_4_-C_12_, whereas most *O adsorption energies approach the mean value of ΔG_*OOH_ and ΔG_*OH_. We further calculated the *OOH, *OH, and *O free energies of adsorption in the theoretical framework of DFT + U. The results were consistent with the DFT calculation (Figure S28 and Table S12). Thus, the different response of *OOH/*O adsorption to the local distortion around the Fe center is caused by phosphorus, which then prompts a higher OER activity of FeN_4_. We further calculated the integrated crystal orbital Hamilton population (ICOHP), which is an effective measure of the bonding strength.^55^ The difference ΔG_*OOH_ – ΔG_*O_ varies linearly with the difference between ICOHP(Fe–OOH) and ICOHP(Fe–O) (Figure 5d). This implies that different responses of OOH/*O adsorption are due to the different binding abilities of *OOH/*O on Fe sites.^23^ In brief, the presence of the phosphorus atom in the second coordination sphere causes a local distortion on the central Fe and tensile strain, which balances the *OOH/*O adsorption characteristics and accordingly facilitates the OER process.

## Conclusions

In summary, we introduce a new single-atom P/Fe–N–C material for rechargeable Zn–air batteries by a phosphorus incorporation protocol. Comprehensive spectroscopic characterization suggests that the phosphorus atom is located in the second coordination sphere of the Fe center. The achieved P/Fe–N–C exhibits excellent OER activity in alkaline media, approaching benchmark IrO_2_, as well as high ORR activity. As a result, the P/Fe–N–C provides state-of-the-art performance as a bifunctional oxygen electrocatalyst for a rechargeable Zn–air battery. According to DFT calculations, the presence of phosphorus efficiently improves the kinetics of O–O formation and breaks the traditional scaling relationship. These results (i) provide a deeper mechanistic understanding of Fe–N–C materials in electrocatalytic oxygen reactions, (ii) are prone to improving the performance in acidic solution by incorporation of heteroatoms, and (iii) will stimulate the search for single-atom catalysts of other electrochemical reactions such as water splitting, CO_2_ reduction, and nitrogen fixation.

## Supporting Information Available

Detailed experimental and characterization methods; schematic of the SAC with first and second coordination spheres; morphologies and crystalline structures of Fe–N–C, P/Fe@N–C, and P/Fe–N–C before and after pyrolysis; Raman spectra; N_2_ adsorption/desorption isotherms; XPS analysis; EXAFS fitting results; OER Tafel plot; EIS analysis; OER performance comparison; CV curves; i–t curve; structural changes after catalytic performance; ORR selectivity analysis and ORR stability; determination of the Fe site density; potential gaps between OER and ORR; possible P-doping Fe–N–C (1-23) and pure Fe–N–C catalysts; Fe-P bonds and formation energy; Fe–N bond lengths and corresponding strain in the P/Fe–N–C material; adsorption energies, free energy changes, overpotential, and the difference of ICOHP; free-energy diagrams; optimal models of possible active sites; correlation between the OER activity and ORR activity; and scaling relation of adsorption energies (PDF)
