# Supplementary material for: Boosting Oxygen Electrocatalytic Activity of Fe–N–C Catalysts by Phosphorus Incorporation
Source: J Am Chem Soc. 2023 Feb 6;145(6):3647–55. doi: 10.1021/jacs.2c12933 (PMC9936543; doi:10.1021/jacs.2c12933)
Supplement: Supplementary file 1 — ja2c12933_si_001.pdf [file ja2c12933_si_001.pdf]

Supporting Information

for

Boosting Oxygen Electrocatalytic Activity of Fe-N-C Catalysts by Phosphorus Incorporation

Yazhou Zhou[1,2,†], Ruihu Lu[3,†], Xiafang Tao[1,2], Zijie Qiu[1,6], Guangbo Chen[4,*], Juan Yang[2], Yan Zhao[3], Xinliang Feng[4,5], Klaus Müllen[1,*]

[1]Max Planck Institute for Polymer Research, 55128 Mainz, Germany

[2]School of Materials Science and Engineering, Jiangsu University, Zhenjiang, Jiangsu 212013, China

[3]State Key Laboratory of Silicate Materials for Architectures, International School of Materials Science and Engineering, Wuhan University of Technology, Wuhan, Hubei 430070, China

[4]Center for Advancing Electronics Dresden (Cfaed) and Faculty of Chemistry and Food Chemistry, Technische Universität Dresden, 01062 Dresden, Germany

[5]Max Planck Institute of Microstructure Physics, Weinberg 2, Halle (Saale), D-06120 Germany

[6]School of Science and Engineering, Shenzhen Institute of Aggregate Science and Technology, The Chinese University of Hong Kong, Shenzhen, Guangdong 518172, China

This file includes:

Methods
Figures. S1 to S28
Tables S1 to S12
References

Methods.

Preparation of P/Fe-N-C. 60 mg of $Fe(NO_3)_3 \cdot 9H_2O$ was dissolved in 200 mL methanol. After the solution was heated to 60 °C, 84 µL of phytic acid (PA, 50 % (w/w) in H_2O) was injected into the above solution with vigorous stirring for 2 h. Afterward, methanol solutions containing 2.5 g of $Zn(NO_3)_2 \cdot 6H_2O$ (200 mL) and 3.0 g of 2-methylimidazole (200 mL) were added sequentially. The mixture was stirred for 24 h at 60 °C. After that, the precipitates, named PA@Fe-ZIF-8, were centrifuged, washed, and dried. The PA@Fe-ZIF-8 powder was milled and then pyrolyzed in the tube furnace at 1000 °C for 1 h under an argon atmosphere. The heating rate and gas flow rate were set to be 35 °C min^{-1} and 50 mL min^{-1}, respectively.

Preparation of P/Fe@N-C. The P/Fe@N-C was synthesized using the same method as P/Fe-N-C, while 112 µL of PA and 90 mg of $Fe(NO_3)_3 \cdot 9H_2O$ were utilized.

Preparation of Fe-N-C. 60 mg of $Fe(NO_3)_3 \cdot 9H_2O$ and 2.5 g of $Zn(NO_3)_2 \cdot 6H_2O$ were dissolved in a 200 mL methanol solution. 200 mL methanol with 3.0 g of 2-methylimidazole were then added to the above mixture. After a 24 h reaction at 60 °C, Fe-ZIF-8 powder was collected by centrifuging and washing processes. Fe-N-C sample was obtained using the same thermal treatment as P/Fe-N-C.

Physical characterization. PXRD measurements were performed on a Rigaku SmartLab diffractometer with Cu Kα X-rays (λ = 1.5406 Å) and a scanning speed of 0.1° min^{-1}. XPS data were collected on Axis Ultra DLD imaging XPS using hybrid mode (700 x 300 µm) with 80-pass energy for survey spectra, as well as 20-pass energy for high-resolution spectra of elements. The Fe content was tested by ICP-OES (VISTA MPX, Varian Inc.). The porous structure was analyzed by N_2 adsorption/desorption experiments that were conducted on a Quantachrome SI-MP Instrument. Non-local density functional theory model was used to determine the pore size distributions. TEM and HR-TEM, together with element mapping images were acquired on Tecnai G2 F30 S-Twin (FEI, Netherlands) working at 200 kV. HAADF-STEM and atomic-resolution-HAADF-STEM images were collected using Theims Z field emission electron microscope (FEI, Netherlands) working at 200 kV. Raman spectra were measured on a Bruker RFS 100/S Raman spectrometer excited by a 532 nm laser. X-ray absorption data was carried out at the 1W1B station of the Beijing Synchrotron Radiation Facility (BSRF) which was operated at 2.5 GeV with a maximum current of 250 mA. Data reduction, analysis, and EXAFS fitting were performed using the ATHENA module implemented in the IFEFFIT software packages according to the standard procedures.

Electrochemical measurements. All the electrochemical measurements were implemented on a CHI 760E electrochemical workstation with the three-electrode system at room temperature. The catalytic ink was prepared by mixing 2.5 mg catalyst, 480 µL of ethanol, and 20 µL of Nafion solution (5 wt%) and vigorous stirring overnight. A certain amount of the catalyst suspension was drop-cast onto a freshly polished glassy carbon rotating disk electrode (RDE, 5.0 mm diameter) or rotating ring disk electrode (RRDE, 5.61 mm diameter, a Pt ring: 6.25 mm inner diameter, 7.92 mm outer diameter), leading to the formation of the uniform film. The catalyst loadings were 0.6 mg cm^{-2} for ORR and 0.4 mg cm^{-2} for OER. Hg/HgO (1 M NaOH) electrode and carbon rod were utilized as reference and counter electrode, respectively. A state-of-the-art Pt/C (20 wt% Pt, Fuelcellstore) was employed as a comparison with a loading of 0.1 mg cm^{-2}. For the OER test, the LSV polarization curves were recorded at 2 mV s^{-1} with a rotating rate of 1600 r.p.m. in O_2 saturated 0.1 M KOH solution. For the ORR test, the LSV polarization curves were recorded at 5 mV s^{-1} with a

rotating rate of 1600 r.p.m. in O_2 saturated 0.1 M KOH solution. Specifically, the OER and ORR reaction pathways were determined by detecting the formation of HO_2^-, the ring electrode was kept at a constant potential of 1.5 V vs RHE in the OER or ORR potential region at a rotating speed of 1600 r.p.m. All potentials were transformed to RHE.

The TOF value of samples towards OER is calculated by equation (1):

$$\mathrm{TOF} = \frac{j \times \mathrm{A}}{4 \times \mathrm{F} \times \mathrm{m}} \qquad (1)$$

where j (A cm^{-2}) is the current density, A (cm^{-2}) is the geometric surface area of the electrode, F (s A mol^{-1}) is the Faraday constant, and m (mol) is the number of moles of metal on the electrode.

Electrochemical impedance spectroscopy (EIS) measurements were recorded at the applied overpotential of 304 mV for OER with frequency from 0.01 Hz to 100 kHz at an alternating current voltage amplitude of 5 mV. ECSAs were estimated based on the C_{dl} at non-faradaic potentials. By plotting the difference of current density (J) between the anodic and cathodic sweeps (J_{anodic} - $J_{cathodic}$) at 1.35 V against the scan rate, a linear trend was observed. Accordingly, the C_{dl} value was obtained according to the equation:

$$C_{dl} = (J_{anodic} - J_{cathodic})/2 \qquad (2)$$

Quantification of the active sites for ORR. The site densities of Fe active sites towards ORR in the Fe-N-C and P/Fe-N-C electrocatalysts were determined according to the method described by Kucernak et al.[1] The method is based on the adsorption and reduction of nitrite (NO_2^-) on the central Fe atoms. The site density and TOF were calculated using the following equations (2 and 3).

$$SD\ (\mathrm{site\ g^{-1}}) = \frac{Q_{strip}\ (C\ g^{-1})}{n_{strip}\ \times\ F\ (C\ mol^{-1})} \qquad (3)$$

$$TOF\ (s^{-1}) = \frac{n_{strip} \Delta j_k\ (\mathrm{mA\ cm^{-2}})}{Q_{strip}(C\ g^{-1}) L_C (\mathrm{mA\ cm^{-2}})} \qquad (4)$$

where Qstrip (C g^{-1}) is the excess coulometric charge associated with the stripping peak, the F is the Faraday constant (F = 96485 C mol^{-1}), and n_{strip} is the number of electrons associated with the reduction of one nitrite per site (n_{strip} = 5), j_k (mA cm^{-2}) is the kinetic current density, $j_k = \frac{j_{lim} \times j}{j_{lim} - j}$, Δjk = ($j_k$(unposioned) – j_k (posiopmed)), m_{cat}, (g) is the mass of catalyst. L_{cat} is the catalyst loading during the reversible nitrite poisoning experiments (0.27 mg cm^{-2}).

Zn-air battery. A home-built electrochemical cell was chosen to study the Zn-air battery performance of the P/Fe-N-C. The catalytic ink was loaded on carbon fiber paper (1 cm^2) with a loading density of 1 mg cm^{-2}. This carbon fiber paper and polished Zn foil were used as the air cathode and anode, respectively. A 6.0 M KOH aqueous solution containing 0.2 M $Zn(OAc)_2$ was employed as the electrolyte solution. All data were recorded from this cell on a Land CT2001A system at room temperature.

Computational details. Spin-polarized density functional theory (DFT) calculations were performed by using ab initio simulation package (VASP).[2-4] The generalized gradient approximation in the Perdew–Burke–Ernzerhof functional was adopted to describe the electron exchange and correlation energy,[5] and the frozen-core projector-augmented wave method with a cutoff energy of 500 eV was chosen to describe the interaction between core electrons and valence electrons.[6, 7] The long-range vdw interactions between atoms is finely described by DFT-D3 correction method in Grimme's scheme.[8] the criteria of energy and force convergence are set to 1.0×10^{-5} eV per atom and 0.02 eV $Å^{-1}$, respectively, for

geometry optimization. And a Γ-centred Monkhorst–Pack k-point mesh grid of 3 × 3 × 1 was employed for all structural optimizations.[9] The vacuum space was 15 Å to avoid artificial interactions between periodic images in z direction.

The OER process is divide into the four fundamental reactions as following:

$$H_2O + * = *OH + H^+ + e^- \quad (5)$$

$$*OH = *O + H^+ + e^- \quad (6)$$

$$*O + H_2O = *OOH + H^+ + e^- \quad (7)$$

$$*OOH = O_2 + H^+ + e^- \quad (8)$$

where, OOH*, O* and OH * present the OOH, O and OH moieties on the adsorption site, respectively. The adsorption energy ($\triangle E_{ads}$) of the key ORR intermediates, including *OOH, *O and *OH, was calculated relative to H_2O and H_2 under conditions of T = 298.15 K, pH = 0, and U = 0 V (*vs*. SHE) according to following equations:

$$\triangle E_{*OOH} = E_{*OOH} + 3/2\, E_{H2} - E_{*} - 2\, E_{H2O} \quad (9)$$

$$\triangle E_{*O} = E_{*O} + E_{H2} - E_{*} - E_{H2O} \quad (10)$$

$$\triangle E_{*OH} = E_{*OH} + 1/2\, E_{H2} - E_{*} - E_{H2O} \quad (11)$$

where * represents adsorbed sites associated with Fe atoms. The above $\triangle G_{ads}$ is defined as the reaction free energies of the following reactions.

$$* + 2H_2O \rightarrow *OOH + 3/2H_2 \quad (12)$$

$$* + H_2O \rightarrow *O + H_2 \quad (13)$$

$$* + H_2O \rightarrow *OH + 1/2H_2 \quad (14)$$

For each element step of OER process, the Gibbs free energies were calculated using the following equation.

$$\Delta G = \Delta E + \Delta E_{ZPE} - T\Delta S \quad (15)$$

where ΔE is the total energy of reactions obtained from DFT calculations. ΔE_{ZPE} and ΔS represent the zero-point energy and entropic changes, respectively, which are obtained *via* vibrational frequencies computations with harmonic approximation and neglecting contributions from the slab. According to the computational hydrogen electrode (CHE) model proposed by Nørskov et al.[10] The free-energy change of $1/2H_2 \rightarrow H^+ + e^-$ reaction is treated to be zero at the potential of 0 and the free energy of proton and electron is set as the $1/2G_{(H2)}$. Because of the difficulties in the DFT calculations of open-shell triple O_2, the free energy of gaseous O_2(g) was calculated by $G_{O2(g)} = 2G_{H2O} - G_{H2} + 4.92$ eV.[11]

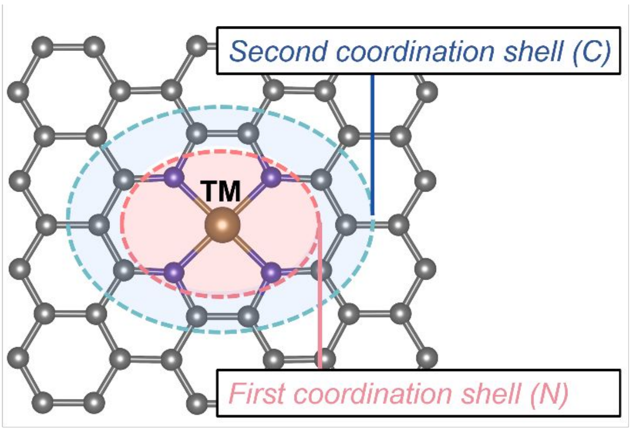

Figure S1. Schematic of FeN_4C_{10} site, highlighting the first and second coordination spheres, where grey, blue, and golden balls represent the C, N, and Fe atoms, respectively.

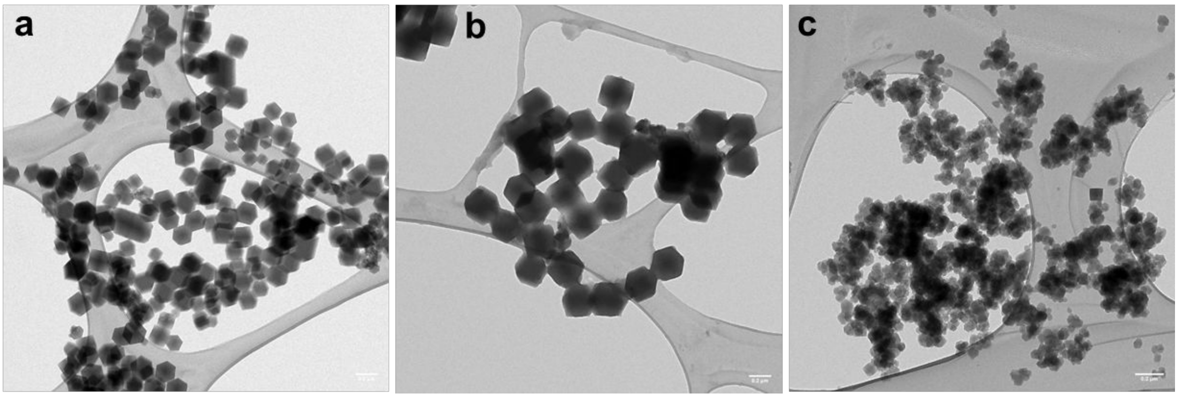

Figure S2. TEM images of precursors for preparation of a) Fe-N-C, b) P/Fe-N-C, and c) P/Fe@N-C.

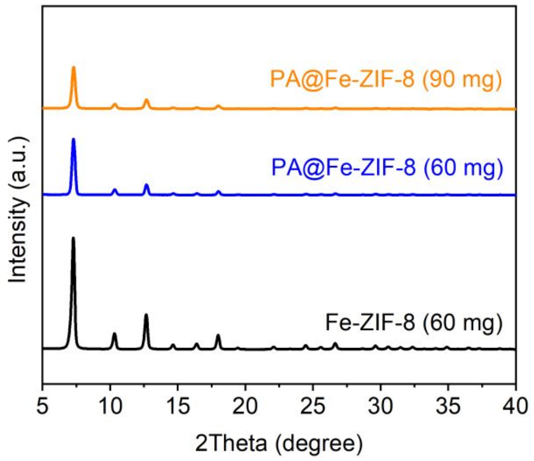

Figure S3. XRD patterns of Fe-ZIF-8, PA@Fe-ZIF-8 (60 mg) and PA@Fe-ZIF-8 (80 mg). The number in the blanket indicates the $Fe(NO_3)_3{\cdot}9H_2O$ mass used in the synthesis process.

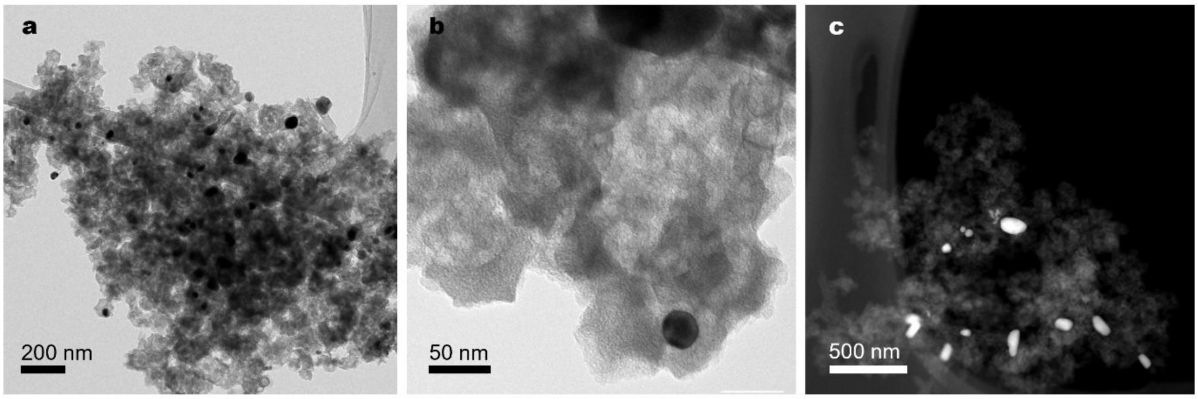

Figure S4. a,b) TEM and c) HAADF-STEM images of P/Fe@N-C.

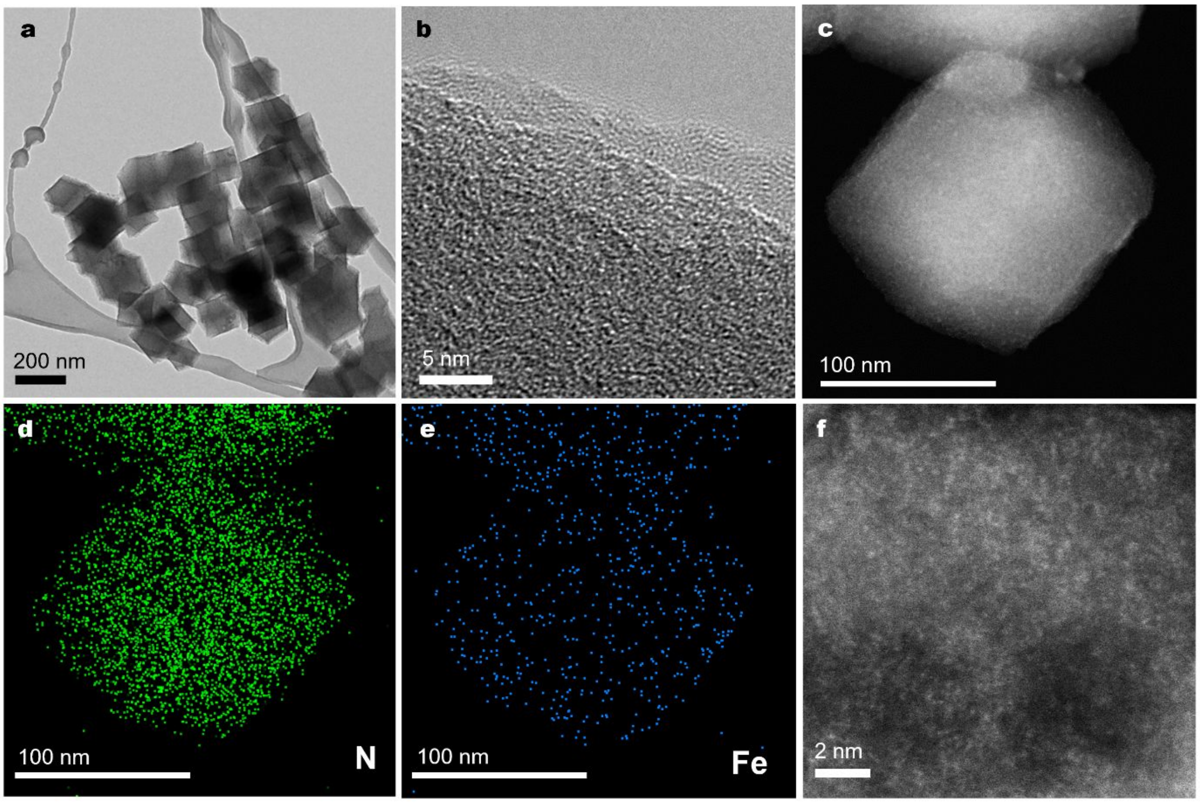

Figure S5. **Characterization of Fe-N-C material.** a) TEM. b) HRTEM. c) HAADF-STEM images, and related elemental mapping images demonstrating the distribution of d) N and e) Fe elements. f) Atomic-resolution HAADF-STEM image.

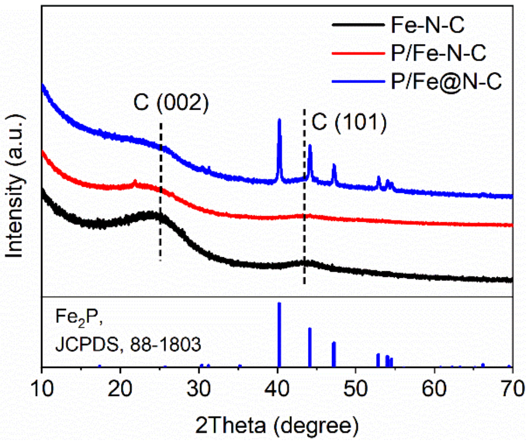

Figure S6. XRD patterns of Fe-N-C, P/Fe-N-C and P/Fe@N-C.

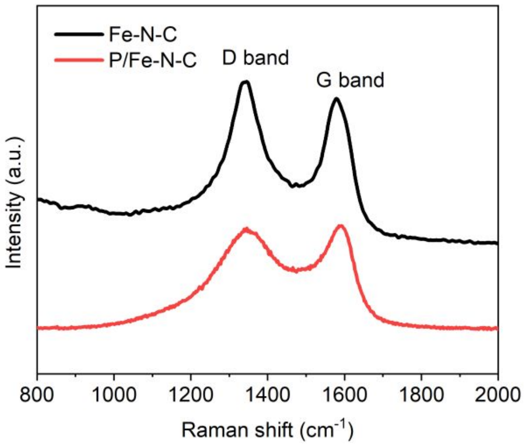

Figure S7. Raman spectra of Fe-N-C and P/Fe-N-C.

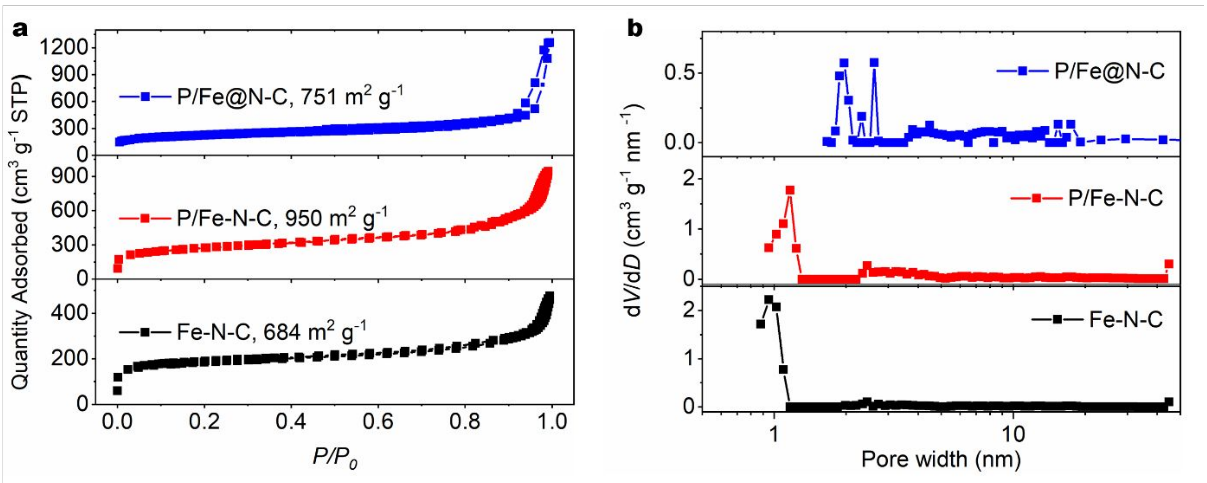

Figure S8. a) N_2 adsorption/desorption isotherms and b) the corresponding pore size distribution curves of Fe-N-C, P/Fe-N-C and P/Fe@N-C.

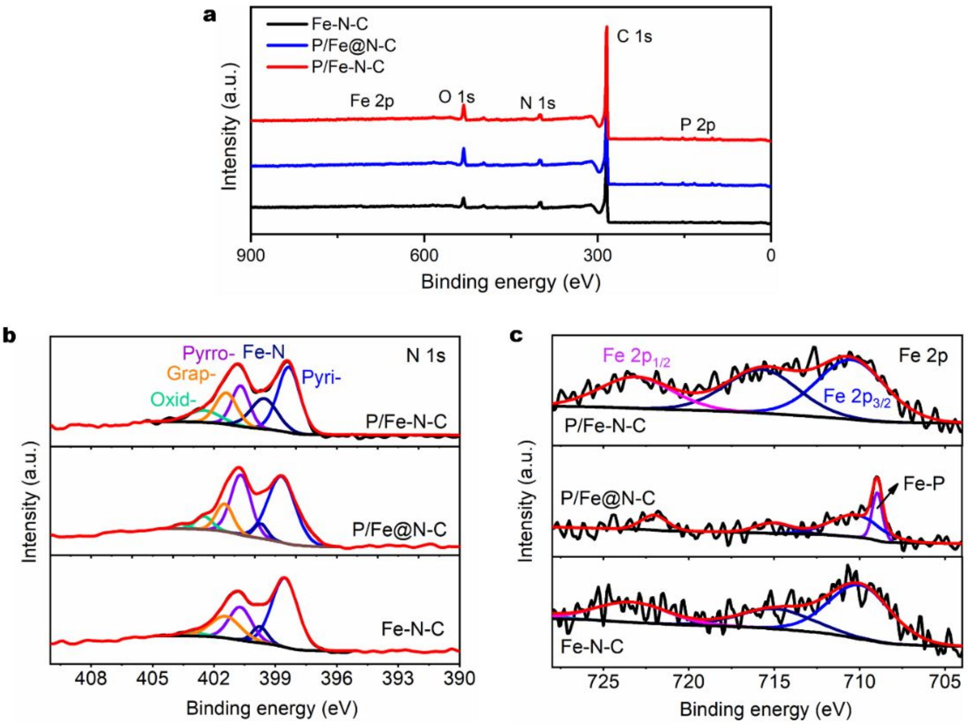

Figure S9. XPS analysis for Fe-N-C, P/Fe-N-C and P/Fe@N-C. a) XPS survey spectra. b) High resolution N 1s XPS spectra. c) High resolution Fe 2p XPS spectra. **The N1** deconvoluted into five N species, including pyridinic-N (398.6 eV), pyrrolic-N (400.5 eV), graphitic-N (401.2 eV), oxidized N (403–405 eV), and Fe-N (399.5 eV).

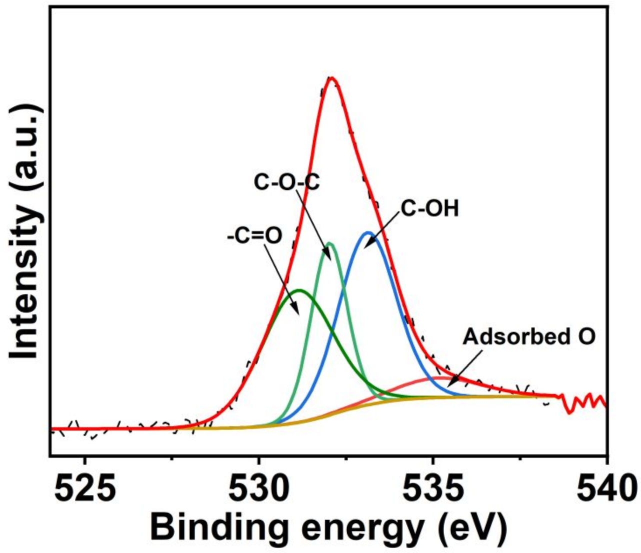

Figure S10. O 1s XPS spectrum of P/Fe-N-C sample

Table S1. Elemental compositions of the Fe-N-C, P/Fe@N-C, and P/Fe-N-C, according to XPS measurements, contents of Fe in samples measured by ICP-OES.

Catalyst	C (at%)	N (at%)	O (at%)	P (at%)	Fe (at%)	Zn (at%)
Fe-N-C	89.7	4.8	4.6	/	0.2	0.3
P/Fe@NC	89.4	3.6	5.4	1.0	0.6	0.2
P/Fe-N-C	90.0	4.0	5.3	0.8	0.4	0.2

Table S2. Fitting results for N 1s spectra of the Fe-N-C, P/Fe@N-C, and P/Fe-N-C.

Catalyst	**Pyridinic N (%)** at ~398.4 eV	**Pyrrolic N (%)** at ~400.5 eV	**Graphitic N (%)** at ~401.6 eV	**FeN_x (%)** at ~399.4 eV	**NO_x (%)** at 402-404 eV
SA-Fe-NC	50.7	21.2	18.6	7.0	2.5
P/Fe@NC	47.9	29.0	11.4	4.7	7.0
P/Fe-N-C	36.0	17.8	16.6	19.9	9.7

Table S3. Structural parameters of the reference materials FeO, Fe_2O_3 and Fe foil and the catalysts Fe-N-C, P/Fe@NC, and P/Fe-N-C extracted from the EXAFS fitting (S_0^2=0.85).

	**Path**	**CN[a]**	**ΔE(eV)[b]**	**R(Å)[c]**	**σ^2(Å^2)[d]**	**R-factor[e]**
Fe foil	Fe-Fe	8	4.9 (1.1)	2.46 (0.01)	0.0045	0.0053
	Fe-Fe1	6		2.84 (0.01)		
FeO	Fe-O	6	-1.83 (2.13)	2.12 (0.02)	0.014	0.009
	Fe-Fe	12	-2.61 (1.10)	3.06 (0.01)	0.011	
Fe_2O_3	Fe-O	6	7.51 (3.22)	1.96 (0.02)	0.011	0.018
	Fe-Fe	6	0.96 (2.31)	2.98 (0.02)	0.0089	
SA-Fe-NC	Fe-N	3.9 (0.9)	4.3 (2.7)	2.06 (0.02)	0.0093	0.013
P/Fe@NC	Fe-N	4.4 (0.8)	1.2 (1.7)	2.03 (0.01)	0.0100	0.0056
	Fe-P	2.0 (0.2)		2.34 (0.02)	0.0047	
	Fe-Fe	2.0 (0.3)		2.70 (0.01)	0.0037	
P/Fe-N-C	Fe-N	4.1 (0.5)	5.5 (3.1)	1.97 (0.02)	0.0056	0.0056
	Fe-P	2.2 (0.7)		2.32 (0.04)	0.0015	

[a]*CN*: coordination numbers; [b]ΔE_0: the inner potential correction.[c]*R*: bond distance; [d]σ^2: Debye-Waller factors; [e]*R* factor: goodness of fit. S_0^2 was set to 0.66, according to the experimental EXAFS fit of Fe foil reference by fixing CN as the known crystallographic value; δ: percentage.

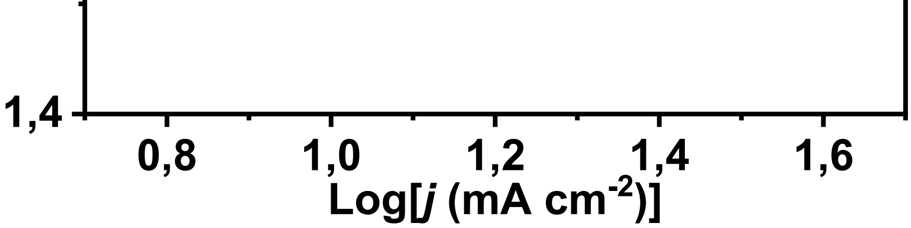

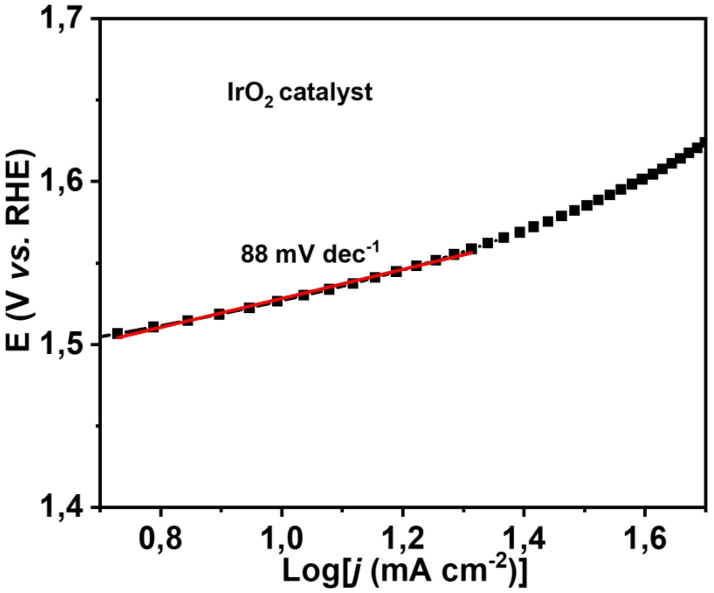

Figure S11. OER Tafel plot of IrO_2 catalyst.

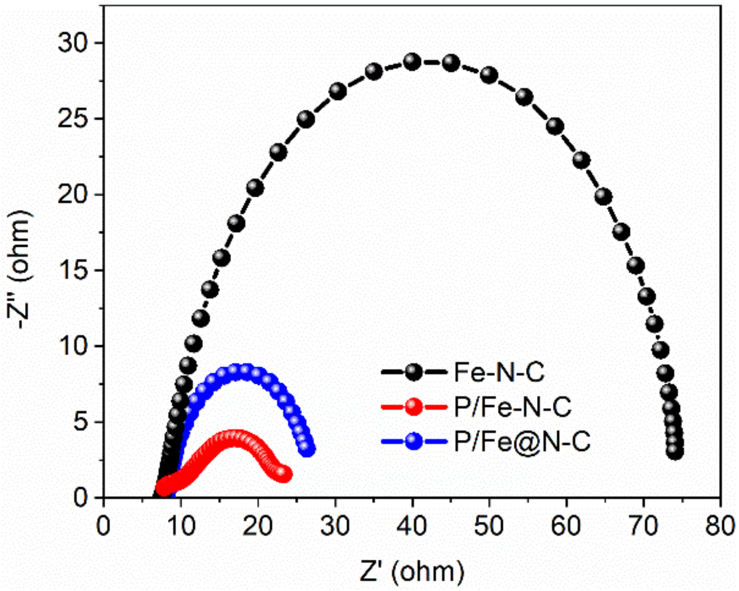

Figure S12. EIS analysis of Fe-N-C, P/Fe-N-C and P/Fe@N-C for OER. EIS measurements were conducted in Ar-saturated 0.1 M KOH aqueous solution at 1.54 V vs. RHE with 5 mV AC potential from 10 kHz to 0.01 Hz. The high frequency interception of the Re-axis represents the resistance of the electrodes and the width of the semicircle on the Re-axis corresponds to the charge-transfer resistances, indicating the overall kinetic effects. Clearly, three electrocatalysts exhibited similar intrinsic resistance, while the charge-transfer resistance of the P/Fe-N-C was much lower than that of the Fe-N-C and P/Fe@N-C, suggesting a faster OER kinetic process on the P/Fe-N-C electrocatalyst.

Table S4. OER performance comparison of P/Fe-N-C and reported state-of-the-art electrocatalysts in alkaline electrolytes.

**Type**	**Catalyst**	**Electrolyte**	**Overpotential at j_{10} (mV)**	**Ref.**
Single atom catalysts	P/Fe-N-C	0.1 M KOH	304	This work
	Fe-N-C	0.1 M KOH	450	This work
	Fe-N_x-C	0.1 M KOH	600	[12]
	S,N-Fe/N/C-CNT		370	[13]
	Ni-NHGF	1.0 M KOH	331	[14]
	Ni-N_4/GHSs/Fe-N_4	0.1 M KOH	390	[15]
	Fe-Ni-N-P-C	1.0 M KOH	337	[16]
	Fe,Mn/N-C	0.1 M KOH	390	[17]
Metal oxides	Co_3O_4/Co-Fe oxide	1.0 M KOH	~340	[18]
	Co-TiO_2		332	[19]
	$Ba_4Sr_4(Co_{0.8}Fe_{0.2})_4O_{15}$		340	[20]
	Mn-Co oxyphosphide		320	[21]
Metal nitrides	$NiCo_2N$		289	[22]
	Mn_3N_2		390	[23]
	Ni_2Mo_3N		270	[24]
	SOA-Co_4N NW/CC		257	[25]
Metal phosphides	Ni_2P nanowires		290	[26]
	NiCoP/C nanoboxes		330	[27]
	MnCoP nanoparticles		330	[28]
	NiCoP/C nanoboxes		330	[27]
	Co_xP		399	[29]
	2D $Co_{1.5}Fe_{0.5}P$		278	[30]

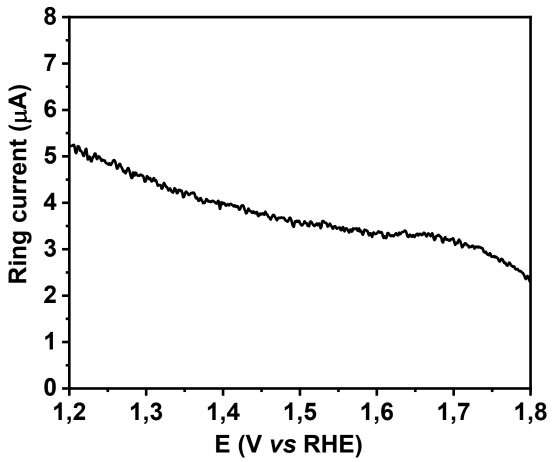

Figure S13. Ring current at E_{ring} = 1.5 V vs RHE of P/Fe-N-C on an RRDE (1600 rpm) in 0.1 M KOH solution when the disk potential is swept in the OER region.

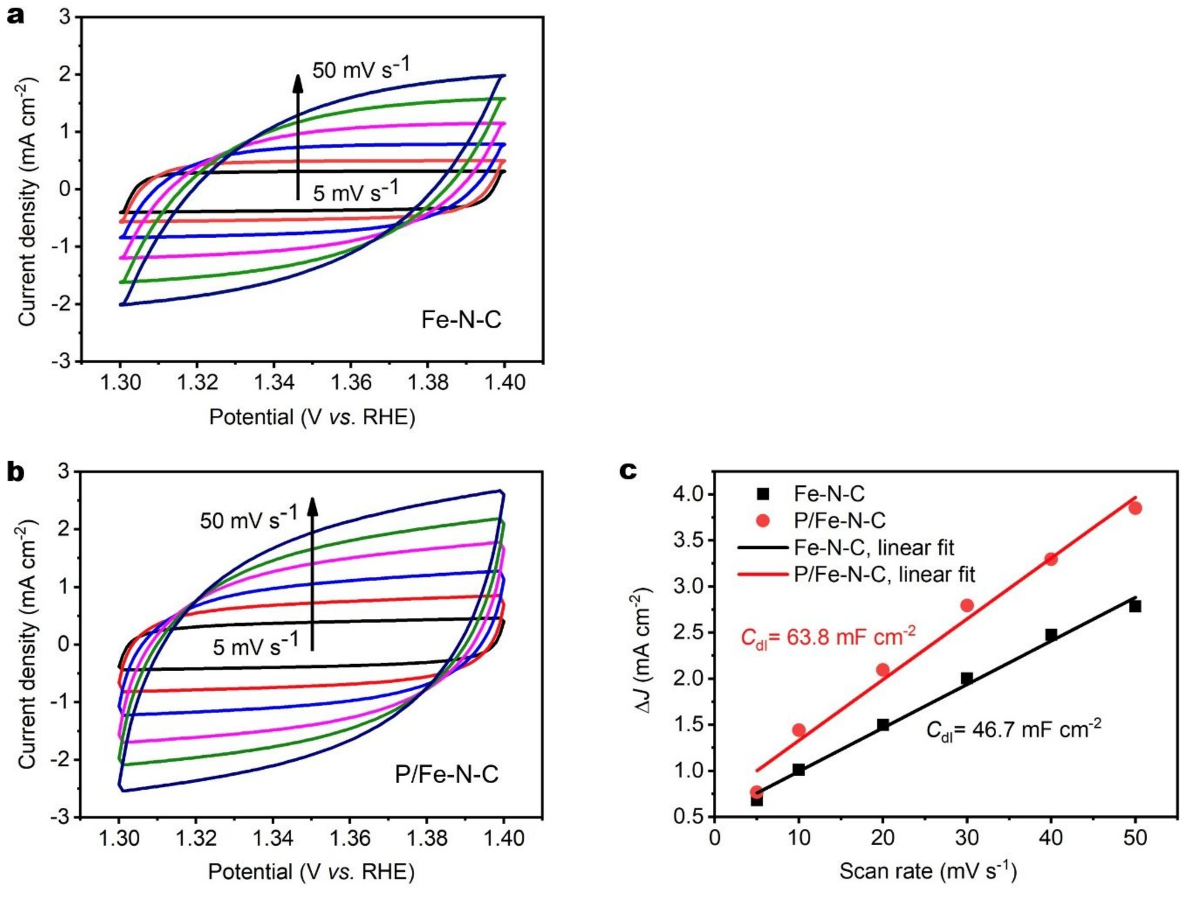

Figure S14. CV curves of a) Fe-N-C, b) P/Fe-N-C modified electrodes in the double layer region at scan rates of 5, 10, 20, 30, 40 and 50 mV s^{-1} in 0.1 M KOH. c) The differences in the current densities (J_{anodic} - $J_{cathodic}$) plotted against the scan rates. The slope of the fitting line is equal to the geometric double layer capacitance (C_{dl}), which is proportional to the effective electrode surface area of the materials.

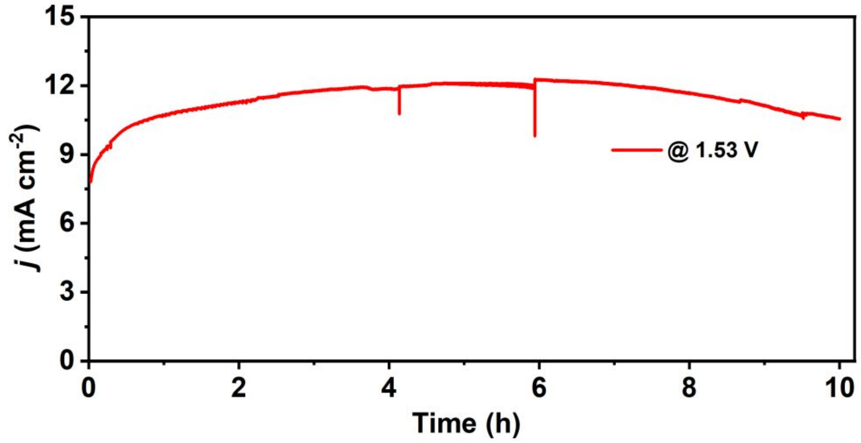

Figure S15. *i–t* curve (where *i* is current density and *t* is time) at constant potentials of 1.53 V for 10 hours.

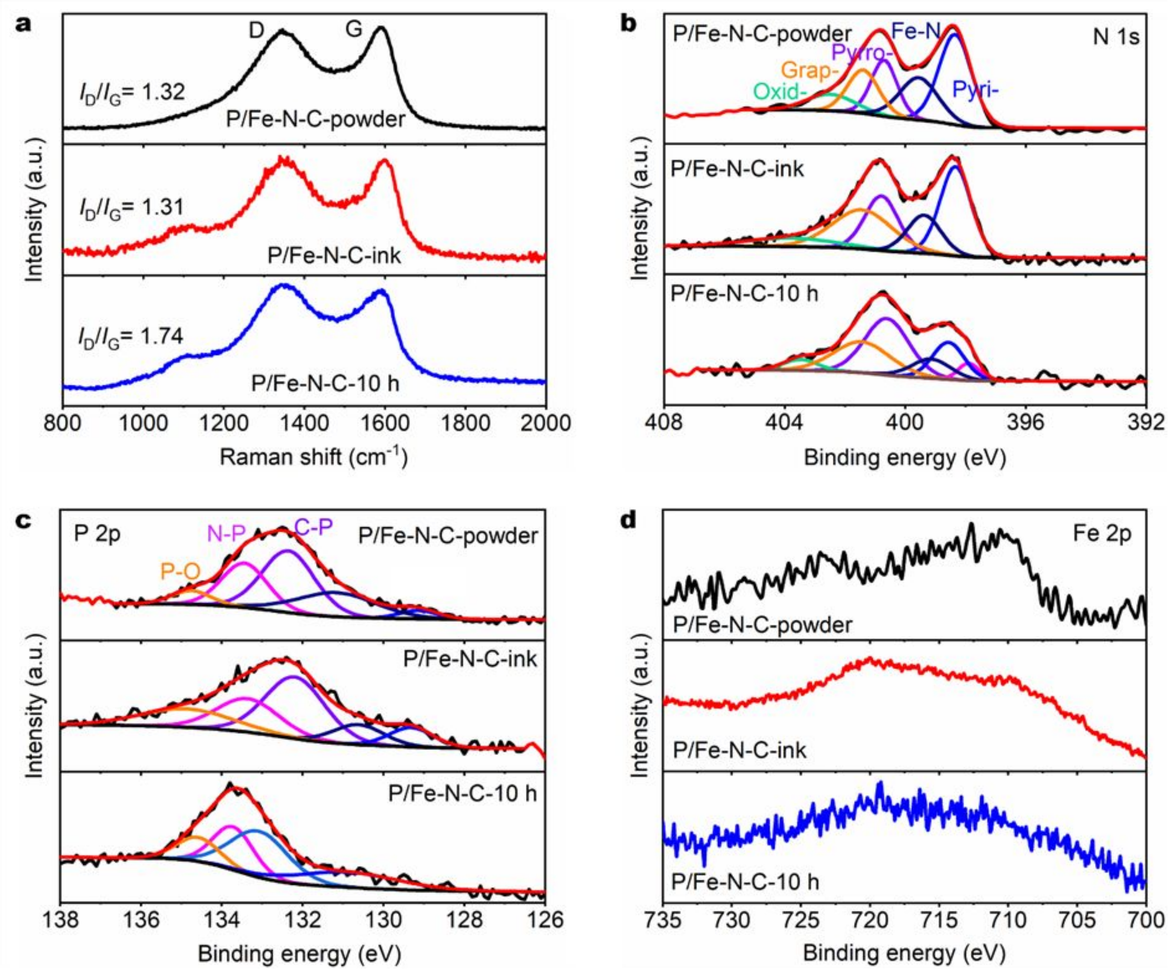

Figure S16. Structural characterization of P/Fe-N-C after the durability OER test holding at constant potential of 1.53 V for 10 hours, a) Raman spectra, b) N 1s, c) P 2p, and d) Fe 2p XPS spectra. These measurements exhibited slightly changed structures of P/Fe-N-C after the durability, including the increase of the defects in carbons, decreased content of pyridinic N, and decreased content of C-P. The Fe structure is nearly same. The slight decreased OER activity is probably due to the reorganized active sites during the durability test.

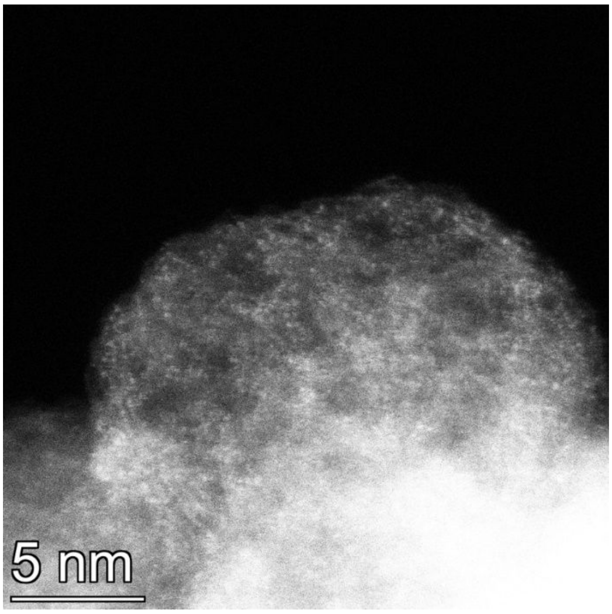

Figure S17. Atomic TEM image of P/Fe-N-C after the after the durability OER test holding at constant potential of 1.53 V for 10 hours. The ignorable Fe aggregates indicate the good stability of P/Fe-N-C.

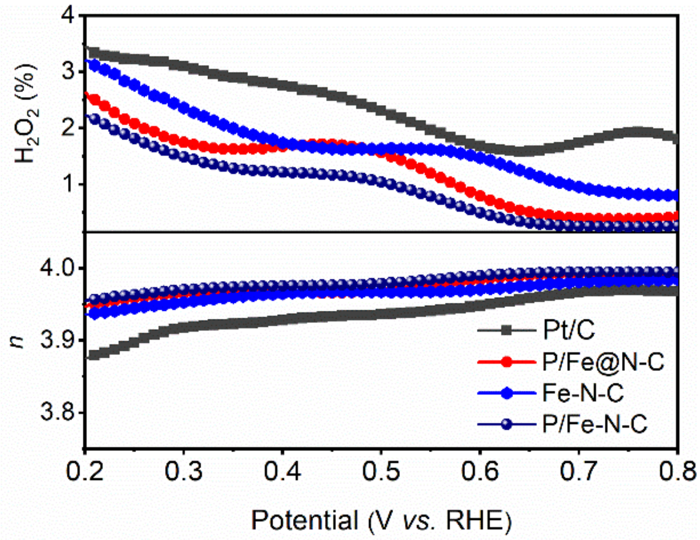

Figure S18. H_2O_2 yield (up) and electron transfer number (below) plots of Fe-N-C, P/Fe@N-C, P/Fe-N-C and Pt/C electrocatalysts.

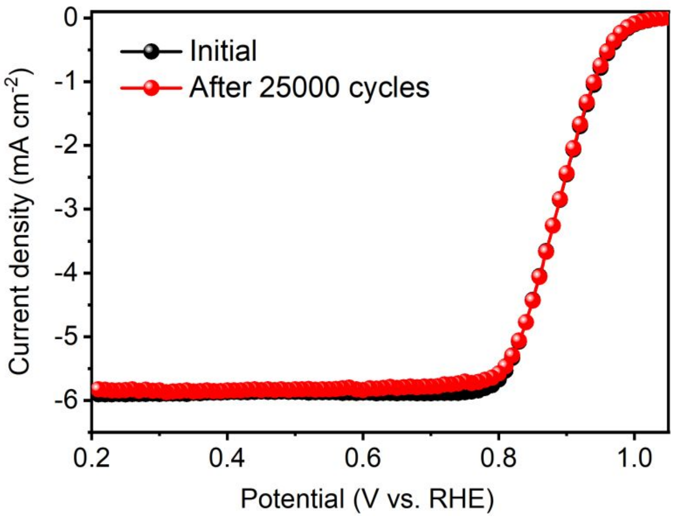

Figure S19. ORR LSV curves before and after 25000 CV cycles.

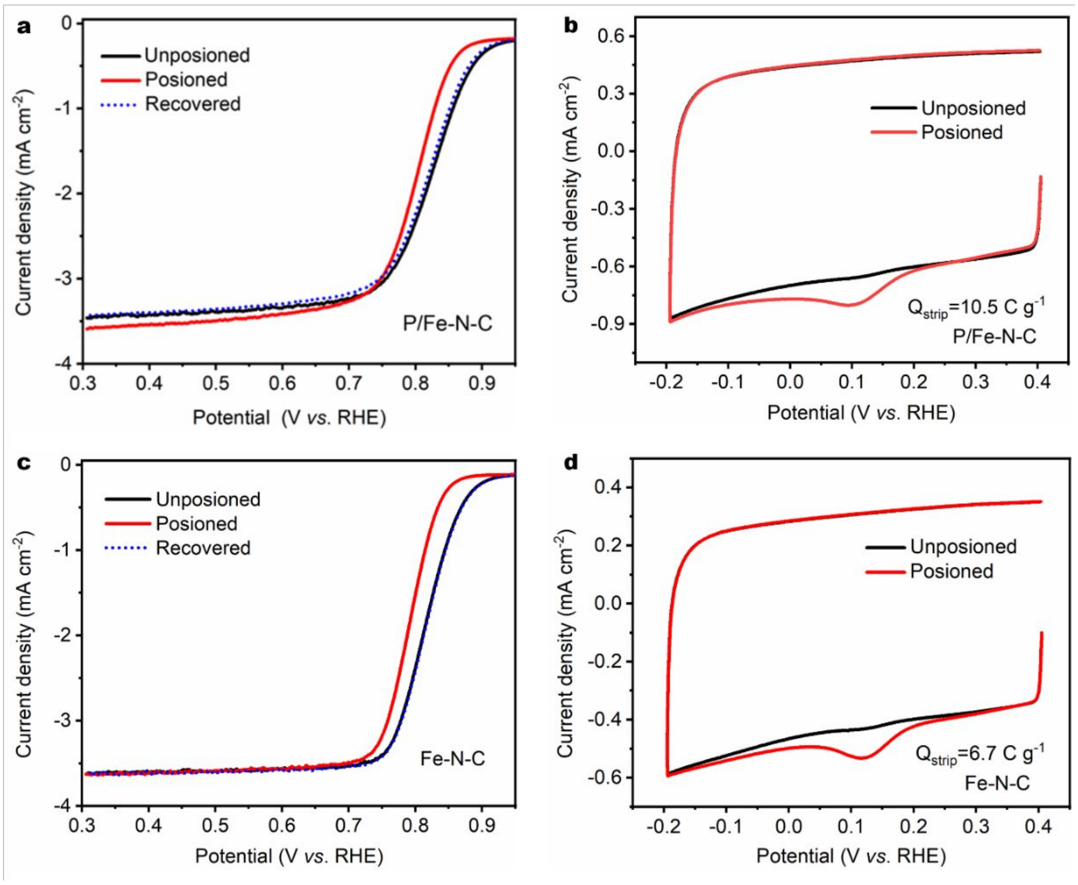

Figure S20. Determination of the Fe site density (SD) for P/Fe-N-C and Fe-N-C. (a) and (c) ORR LSV curves before, during and after nitrite adsorption in an O_2-saturated 0.5 M acetate buffer at pH 5.2. (b) and (d) CV curves before and during nitrite adsorption in the nitrite reductive stripping region. Catalyst loading: 0.27 mg cm^{-2}.

Table S5. TOF at 0.85 V and FeN_4 site densities (SD) for P/Fe-N-C and Fe-N-C catalysts towards ORR.

**Catalyst**	**Method**	**TOF at 0.85V (s^{-1})**	**SD (µmol g^{-1})**
P/Fe-N-C	NO_2^-	2.0	21.8
Fe-N-C	NO_2^-	2.5	13.9

Table S6. Potential gaps (ΔE) between OER and ORR of reported state-of-the-art bifunctional electrocatalysts.

	**Catalyst**	**OER potential at j_{10} (V)**	**$E_{1/2}$ of ORR (V)**	**ΔE (V)**	**Ref.**
Single-atom catalyst	P/Fe-N-C	1.535	0.9	0.635	This work
	Fe-N-C	1.685	0.88	0.805	
	P/Fe@N-C	1.614	0.87	0.744	
	S,N-Fe/N/C-CNT	1.6	0.85	0.75	[13]
	NiN4/GHSs/FeN4	1.62	0.83	0.79	[15]
	Fe,Mn/NC	1.62	0.928	0.692	[17]
	FeNi SAs/NC	1.5	0.84	0.66	[31]
	meso/micro-FeCo-N_x-CN	1.67	0.886	0.784	[32]
	Fe/N-G-SAC	1.6	0.89	0.71	[33]
	Co-N,B-CSs	1.66	0.83	0.83	[34]
Metal-free	N-GRW	1.66	0.84	0.82	[35]
	B,N-carbon	1.57	0.84	0.73	[36]
	NPCSs	1.64	0.83	0.81	[37]
	2D-PPCN	1.595	0.85	0.745	[38]
	GH-BGQD	1.6	0.87	0.73	[39]
	NKCNPs	1.71	0.79	0.92	[40]
Metal oxides	Co_3O_{4-x} HoNPs@HPNCS	1.574	0.834	0.74	[41]
	Co_3O_4-NP/N-rGO	1.61	0.76	0.85	[42]
	Co@Co_3O_4/NC	1.65	0.8	0.85	[43]
	Co_2FeO_4/NCNTs	1.65	0.8	0.85	[44]
	$ZnCo_2O_4$/N-CNT	1.65	0.87	0.78	[45]
Metal nitrides	Ni_3FeN/Co,N-CNF	1.5	0.81	0.69	[46]
	Fe_3Pt/Ni_3FeN	1.595	0.93	0.665	[47]
	Ni-Fe-MoNNTs	1.53	0.72	0.81	[48]
Metal selenides	$NiSe_2/CoSe_2$-N	1.516	0.81	0.706	[49]
	$Ni_xCo_{0.85-x}Se$	1.535	0.78	0.755	[50]
	N-$CoSe_2$/3D MXene	1.54	0.79	0.79	[51]
Perovskites	$Pb_2Ru_2O_{6.5}$	1.6	0.81	0.79	[52]
	$LaNi_{0.85}Mg_{0.15}O_3$	1.68	0.69	0.99	[53]
	$S_{5.84\%}$-$LaCoO_3$	1.594	0.704	0.89	[54]
	Pt-SCFP/C-12	1.6	0.81	0.79	[55]
PGM-based	Pt/C+IrO_2	1.528	0.84	0.688	This work
	SA-PtCoF	1.538	0.88	0.658	[56]
	PdMo bimetallene	1.7	0.95	0.75	[57]
	Pd/FeCo	1.55	0.85	0.7	[58]

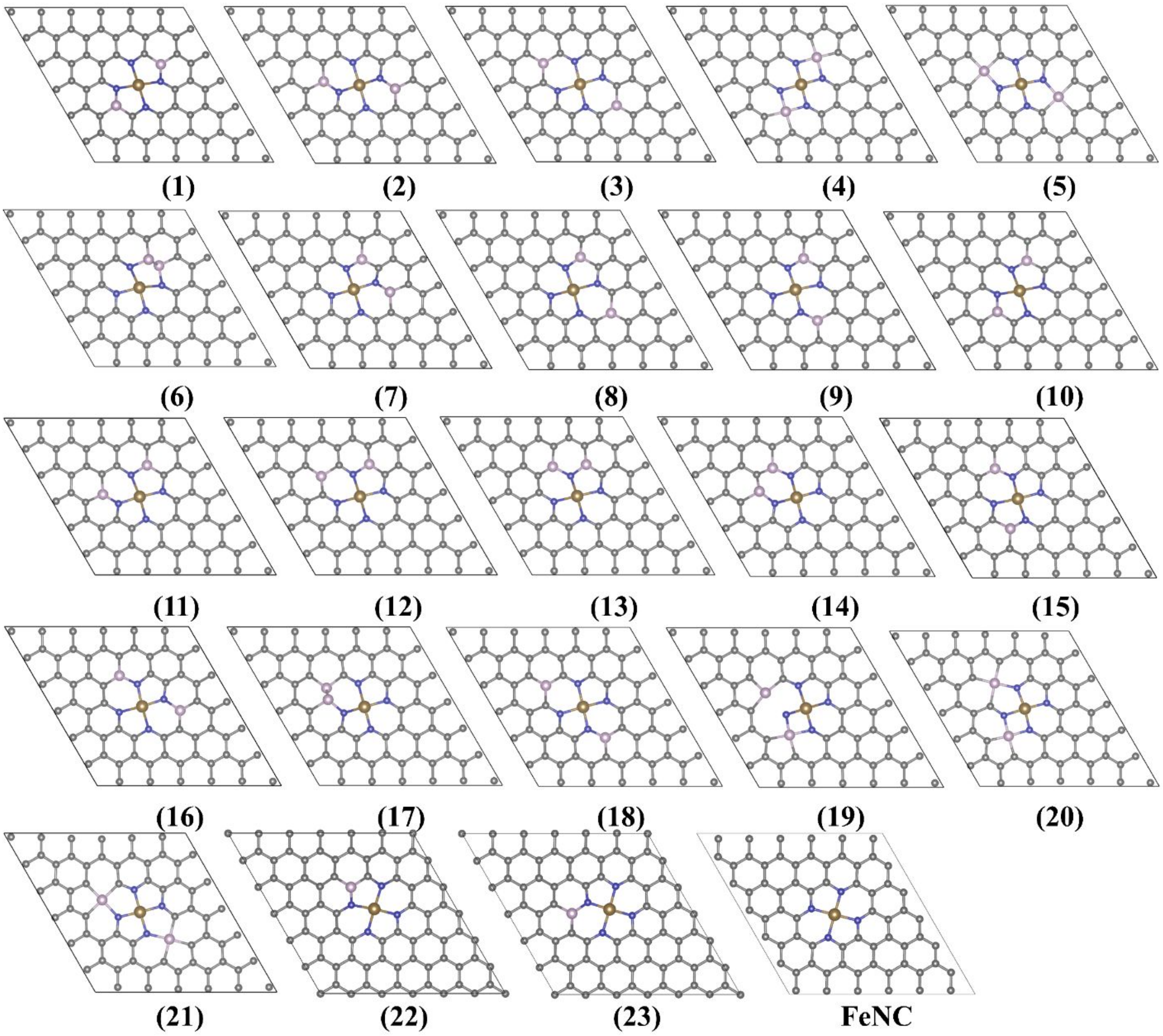

Figure S21. Possible P-doping Fe-N-C (1-23) and pure Fe-N-C catalysts.

Table S7. Fe-P bonds and formation energy of possible P-doping Fe-N-C and pure Fe-N-C catalysts. The gray-labelled structure is chosen to represent P-doping Fe-N-C through the standard of Fe-P_1 or P_2 < 3 Å and E_f < -5 eV.

Structure	Fe-P_1/Å	Fe-P_2/Å	E_f/eV	Structure	Fe-P_1/Å	Fe-P_2/Å	E_f/eV
(1)	2.89	2.89	-8.03	(13)	2.95	3.27	-8.25
(2)	3.18	3.18	-8.16	(14)	3.22	3.22	-8.14
(3)	3.54	3.54	-7.56	(15)	3.24	2.86	-8.06
(4)	2.57	2.57	-4.09	(16)	3.14	3.14	-8.10
(5)	3.22	3.22	-4.59	(17)	3.20	3.55	-8.87
(6)	2.60	2.60	-8.22	(18)	3.53	3.12	-7.87
(7)	3.16	2.89	-8.14	(19)	2.52	3.50	-5.26
(8)	2.89	3.64	-7.43	(20)	3.26	2.64	-5.38
(9)	2.79	3.12	-7.76	(21)	3.25	3.25	-5.58
(10)	2.88	2.88	-7.53	(22)	2.89	/	-5.07
(11)	2.87	3.12	-7.95	(23)	3.15	/	-5.18
(12)	2.95	3.63	-7.30	Fe-N-C	/	/	-2.20

The formation energy (ΔE_f) was calculated as the following equation:

$$\Delta E_{\mathrm{f}} = E_{\mathrm{P/Fe-N-C}} - a\mu_{\mathrm{C}} - b\mu_{\mathrm{Fe}} - c\mu_{\mathrm{N}} - d\mu_{\mathrm{P}}$$

Where $E_{\mathrm{P/Fe-N-C}}$ is he total energy of P/Fe-N-C system. μ_{C}, μ_{Fe}, μ_{N}, and μ_{P} are the chemical potential of C, Fe, N, and P atoms defined as the total energy per atom in the stable elementary substance, such as graphene, Fe metal, N2 molecule, and elemental phosphorus. a, b, c, and d represent the numbers of C, Fe, N, and P atoms in P/Fe-N-C, respectively.

Table S8. Fe-N bond lengths and corresponding strain in the P/Fe-N-C material.

	**Fe-N_1/Å**	**Fe-N_2/Å**	**Fe-N_3/Å**	**Fe-N_4/Å**	**S_1/%**	**S_2/%**	**S_3/%**	**S_4/%**
**Fe-N-C**	1.90	1.90	1.90	1.90				
**(1)**	1.96	1.89	1.96	1.89	3.22	-0.37	3.22	-0.37
**(6)**	1.94	1.94	1.95	1.94	2.37	2.53	2.58	2.43
**(7)**	1.86	1.86	1.99	1.88	-1.74	-1.74	4.69	-0.69
**(9)**	1.86	2.02	1.84	1.91	-1.79	6.54	-3.01	0.69
**(10)**	1.90	1.99	1.99	1.90	0.42	5.12	5.17	0.37
**(11)**	1.95	1.94	1.93	1.83	2.85	2.53	1.79	-3.69
**(15)**	1.85	1.91	1.88	2.01	-2.32	0.47	-0.79	5.80
**(22)**	1.95	1.91	1.92	1.88	2.69	0.79	1.00	-0.63
**(23)**	1.90	1.89	1.94	1.83	0.21	-0.53	2.37	-3.43

Table S9. The adsorption energies (ΔG_{*OOH}, ΔG_{*OH}, and ΔG_{*O}, in unit of eV), free energy changes (ΔG_i (i=1-4), eV), overpotential (η, in an unit of V), and the difference of Integrated Crystal Orbital Hamilton Population (ΔICOHP=ICOHP(Fe-OOH)-ICOHP(Fe-O), in an unit of V) of chosen P-doping Fe-N-C and pure Fe-N-C materials.

Structure	ΔG_{*OOH}	ΔG_{*OH}	ΔG_{*O}	ΔG_1	ΔG_2	ΔG_3	ΔG_4	η	ΔICOHP
Fe-N-C	3.52	0.59	1.37	0.59	0.78	2.15	1.40	0.92	-0.13
(1)	3.62	0.61	1.48	0.61	0.88	2.14	1.30	0.91	1.37
(6)	3.15	-0.07	1.61	-0.07	1.67	1.54	1.77	0.54	2.63
(7)	3.16	0.13	1.49	0.13	1.36	1.67	1.76	0.53	1.94
(9)	2.78	-0.23	1.25	-0.23	1.48	1.53	2.14	0.91	2.10
(10)	2.94	0.24	1.59	0.24	1.35	1.35	1.98	0.75	3.50
(11)	3.16	0.09	1.58	0.09	1.49	1.59	1.76	0.53	2.18
(15)	3.07	0.06	1.52	0.06	1.45	1.55	1.85	0.62	1.93
(22)	3.13	0.12	1.55	0.12	1.44	1.58	1.79	0.56	2.11
(23)	3.05	-0.19	1.32	-0.19	1.50	1.73	1.87	0.64	3.35

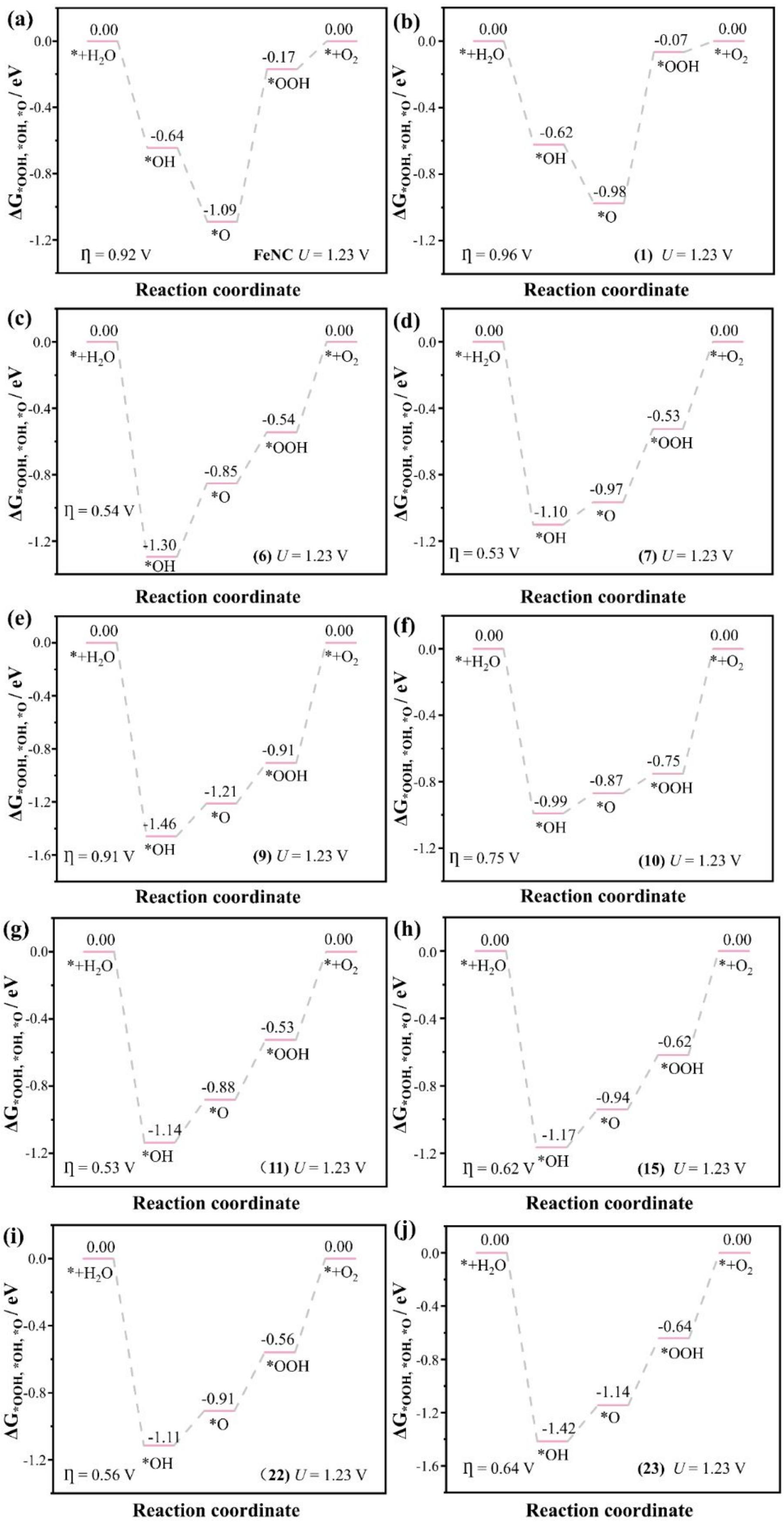

Figure S22. Free-energy diagrams for OER over different structures in P-doping Fe-N-C catalyst.

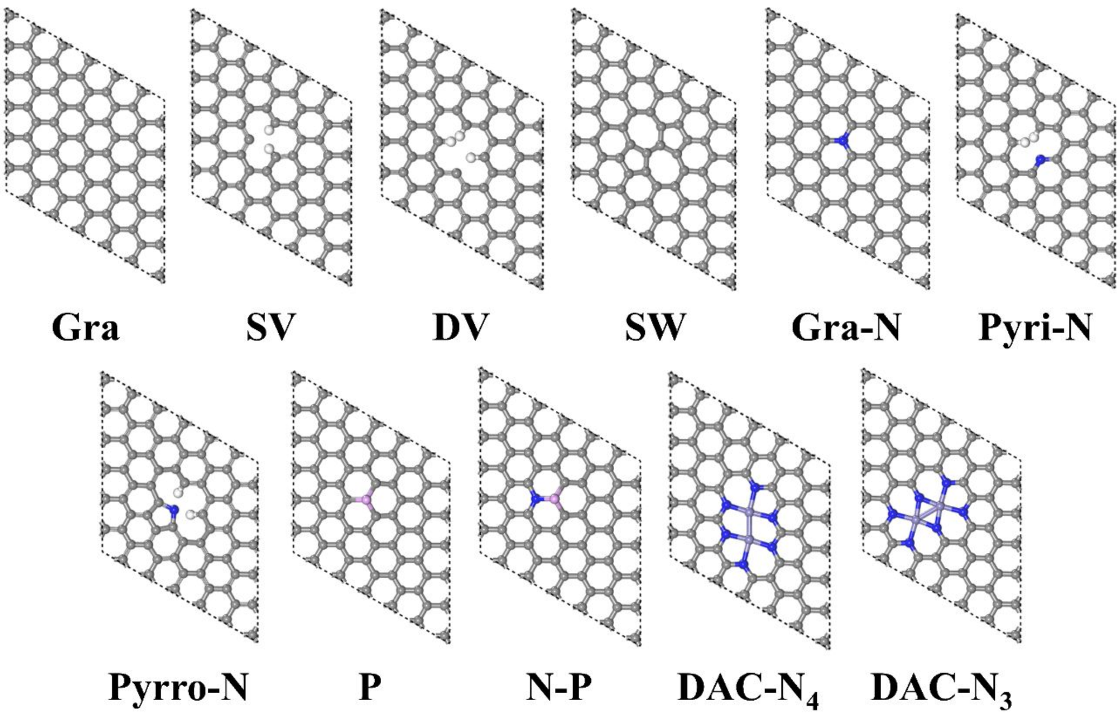

Figure S23. Optimal models of possible active sites in P-doping Fe-N-C catalyst.

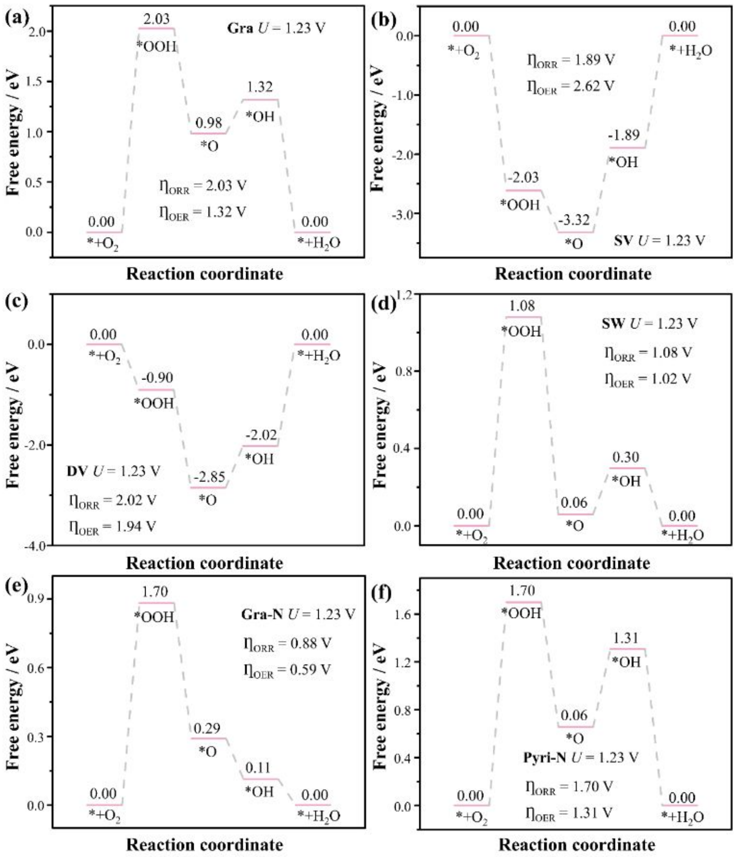

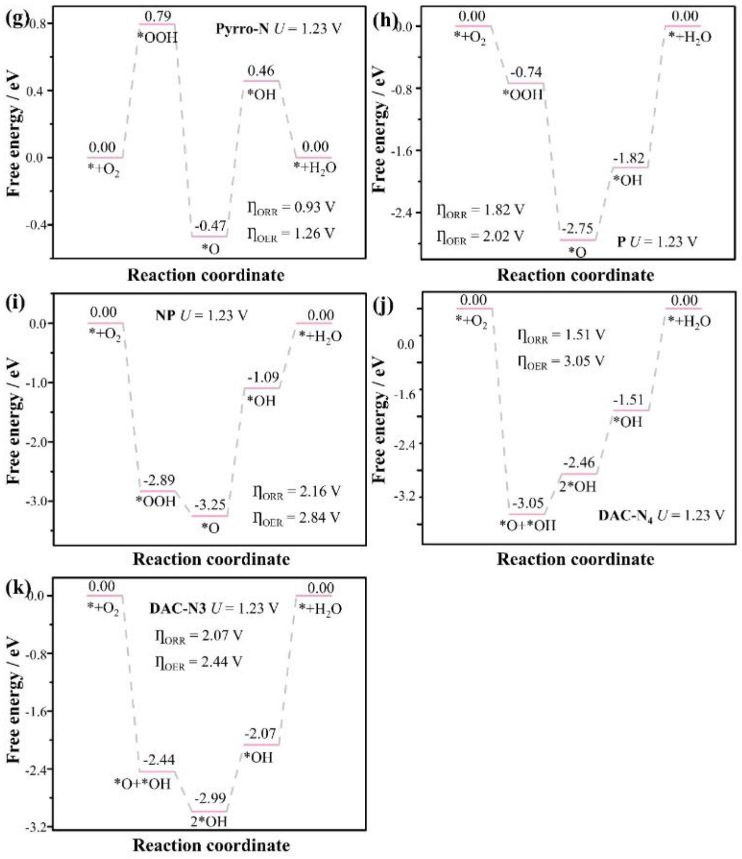

Figure S24. Free-energy diagrams for OER over different structures in P-doping Fe-N-C catalyst.

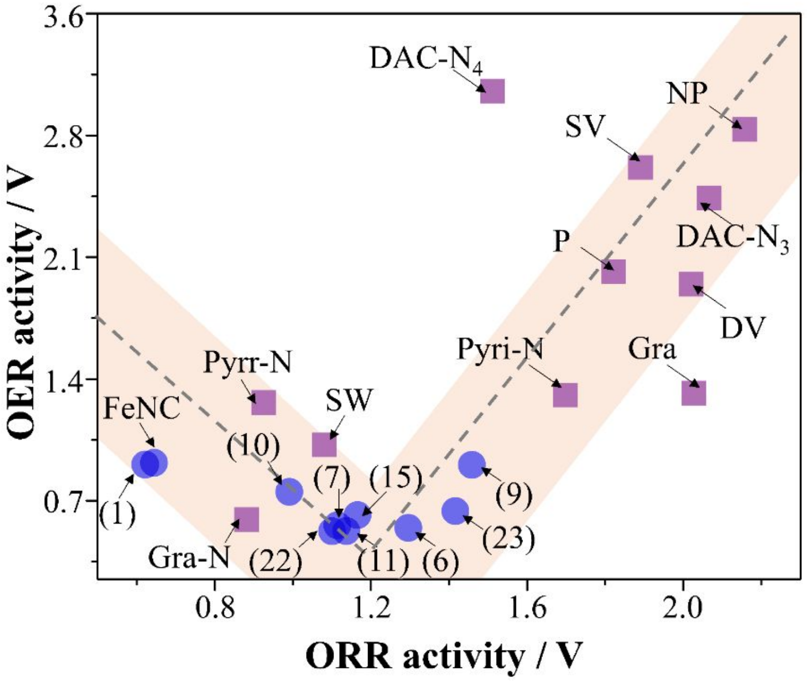

Figure S25. Correlation between the OER activity and ORR activity over different active sites in the P/Fe-N-C catalyst.

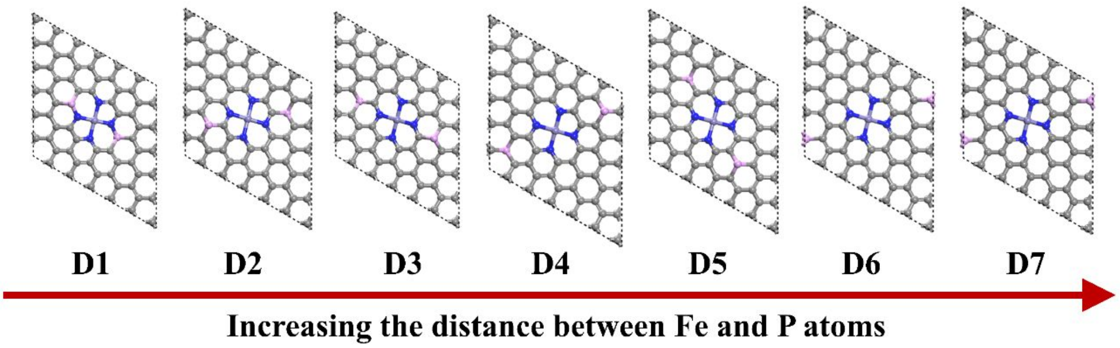

Figure S26. Optimized P-doping Fe-N-C with increasing distance between Fe and P atoms.

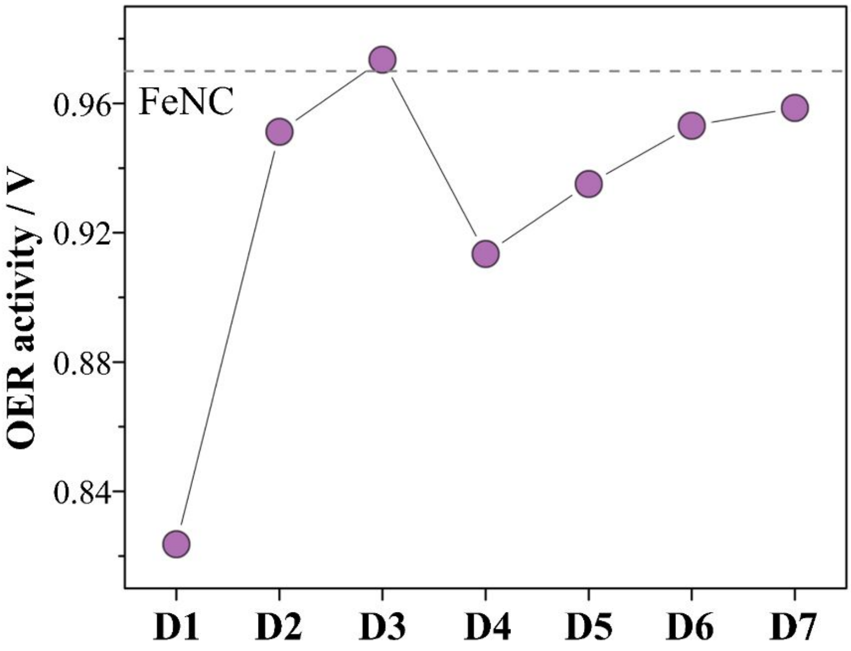

Figure S27. The OER activity of P-doping Fe-N-C with increasing distance between Fe and N atoms. The dotted line denotes the OER overpotential of pure Fe-N-C.

Table S10. Fe-N bond lengths and corresponding strain in the P/Fe-N-C material.

	$Fe\text{-}N_1$/Å	$Fe\text{-}N_2$/Å	$Fe\text{-}N_3$/Å	$Fe\text{-}N_4$/Å	S_1/%	S_2/%	S_3/%	S_4/%
**Fe-N-C**	1.90	1.90	1.90	1.90				
**(1)**	1.96	1.89	1.96	1.89	3.22	-0.37	3.22	-0.37
**(6)**	1.94	1.94	1.95	1.94	2.37	2.53	2.58	2.43
**(7)**	1.86	1.86	1.99	1.88	-1.74	-1.74	4.69	-0.69
**(9)**	1.86	2.02	1.84	1.91	-1.79	6.54	-3.01	0.69
**(10)**	1.90	1.99	1.99	1.90	0.42	5.12	5.17	0.37
**(11)**	1.95	1.94	1.93	1.83	2.85	2.53	1.79	-3.69
**(15)**	1.85	1.91	1.88	2.01	-2.32	0.47	-0.79	5.80
**(22)**	1.95	1.91	1.92	1.88	2.69	0.79	1.00	-0.63
**(23)**	1.90	1.89	1.94	1.83	0.21	-0.53	2.37	-3.43

Table S11. The free energy change (ΔG_i, i=1-4, in unity of eV) and adsorption energy (ΔG_{*OOH}, ΔG_{*OH}, and ΔG_{*O}, eV) of ORR/OER on P-doped Fe-N-C with increasing distance between Fe and N atoms.

	ΔG_1	ΔG_2	ΔG_3	ΔG_4	ΔG_{*OOH}	ΔG_{*OH}	ΔG_{*O}	η_{ORR}	η_{OER}
	*→*OOH	*OOH→*O	*O→*OH	*O→*					
**Fe-N-C**	-1.36	-2.20	-0.76	-0.60	3.56	0.60	1.36	0.63	0.97
**D1**	-1.34	-2.05	-1.01	-0.52	3.58	0.52	1.53	0.71	0.82
**D2**	-1.39	-2.18	-0.89	-0.46	3.53	0.46	1.35	0.77	0.95
**D3**	-1.29	-2.20	-0.72	-0.70	3.63	0.70	1.43	0.53	0.97
**D4**	-2.01	-2.14	-0.89	0.12	2.91	-0.12	0.77	1.35	0.91
**D5**	-1.36	-2.17	-0.79	-0.61	3.56	0.61	1.39	0.62	0.94
**D6**	-1.31	-2.18	-0.76	-0.66	3.61	0.66	1.42	0.57	0.95
**D7**	-1.32	-2.19	-0.75	-0.66	3.60	0.66	1.41	0.57	0.96

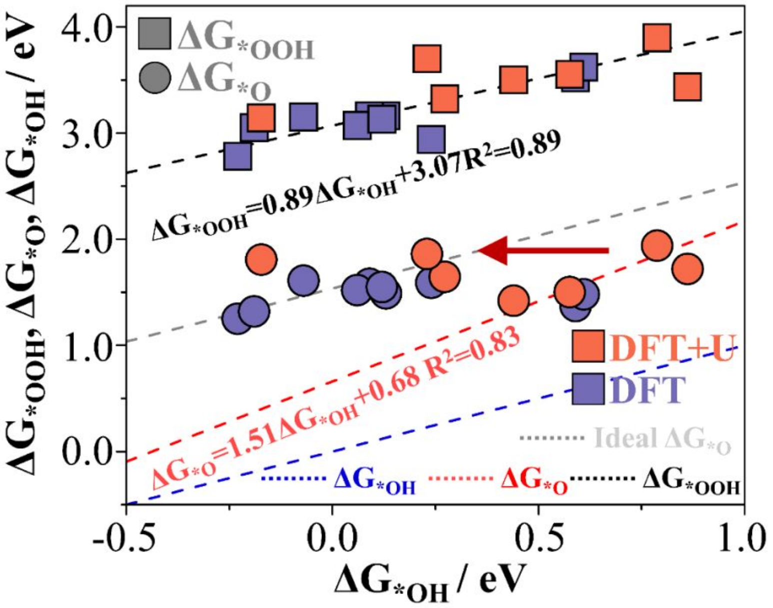

Figure S28. Scaling relation of adsorption energies for different OER intermediates on the P/Fe-N-C. The dotted lines denote the statistics scaling relation on TMN_4C_{12} originated from Figure 5b.

Table S12. The adsorption energy and free energy change of OER on pure and P-doping Fe-N-C

	ΔG_{*OOH}	ΔG_{*OH}	ΔG_{*O}	ΔG_1	ΔG_2	ΔG_3	ΔG_4	η_{OER}
**Fe-N-C**	3.52	0.59	1.37	0.59	0.78	2.15	1.40	0.92
**Fe-N-C-U**	3.55	0.58	1.50	0.58	0.93	2.05	1.37	0.82
**(1)**	3.62	0.61	1.48	0.61	0.88	2.14	1.30	0.91
**(1)-U**	3.32	0.27	1.65	0.27	1.37	1.68	1.60	0.45
**(6)**	3.15	-0.07	1.61	-0.07	1.67	1.54	1.77	0.54
**(6)-U**	3.14	-0.17	1.81	-0.17	1.98	1.33	1.78	0.75
**(7)**	3.16	0.13	1.49	0.13	1.36	1.67	1.76	0.53
**(7)-U**	3.43	0.86	1.72	0.86	0.86	1.71	1.49	0.48
**(9)**	2.78	-0.23	1.25	-0.23	1.48	1.53	2.14	0.91
**(9)-U**	4.17	1.10	2.51	1.10	1.41	1.65	0.75	0.42
**(10)**	2.94	0.24	1.59	0.24	1.35	1.35	1.98	0.75
**(10)-U**	4.48	1.37	2.33	1.37	0.96	2.15	0.44	0.92
**(11)**	3.16	0.09	1.58	0.09	1.49	1.59	1.76	0.53
**(11)-U**	3.50	0.44	1.42	0.44	0.98	2.08	1.42	0.85
**(15)**	3.07	0.06	1.52	0.06	1.45	1.55	1.85	0.62
**(15)-U**	4.25	1.19	2.24	1.19	1.04	2.01	0.67	0.78
**(22)**	3.13	0.12	1.55	0.12	1.44	1.58	1.79	0.56
**(22)-U**	3.90	0.79	1.94	0.79	1.15	1.96	1.02	0.73
**(23)**	3.05	-0.19	1.32	-0.19	1.50	1.73	1.87	0.64
**(23)-U**	3.70	0.23	1.86	0.23	1.63	1.84	1.22	0.61

Supplementary References

(1) Malko, D.; Kucernak, A.; Lopes, T., In situ electrochemical quantification of active sites in Fe-N/C non-precious metal catalysts. *Nat. Commun.* **2016,** *7* (1), 13285.

(2) Kresse, G.; Furthmüller, J., Efficiency of ab-initio total energy calculations for metals and semiconductors using a plane-wave basis set. *Comput. Mater. Sci.* **1996,** *6* (1), 15-50.

(3) Kresse, G.; Furthmüller, J., Efficient iterative schemes for ab initio total-energy calculations using a plane-wave basis set. *Phys. Rev. B* **1996,** *54* (16), 11169-11186.

(4) Kresse, G.; Hafner, J., Ab initio molecular dynamics for liquid metals. *Phys. Rev. B* **1993,** *47* (1), 558-561.

(5) Perdew, J. P.; Burke, K.; Ernzerhof, M., Generalized Gradient Approximation Made Simple. *Phys. Rev. Lett.* **1996,** *77* (18), 3865-3868.

(6) Kresse, G.; Joubert, D., From ultrasoft pseudopotentials to the projector augmented-wave method. *Phys. Rev. B* **1999,** *59* (3), 1758-1775.

(7) Blöchl, P. E., Projector augmented-wave method. *Phys. Rev. B* **1994,** *50* (24), 17953-17979.

(8) Grimme, S.; Antony, J.; Ehrlich, S.; Krieg, H., A consistent and accurate ab initio parametrization of density functional dispersion correction (DFT-D) for the 94 elements H-Pu. *J. Chem. Phys.* **2010,** *132* (15), 154104.

(9) Monkhorst, H. J.; Pack, J. D., Special points for Brillouin-zone integrations. *Phys. Rev. B* **1976,** *13* (12), 5188-5192.

(10) Nørskov, J. K.; Bligaard, T.; Logadottir, A.; Kitchin, J. R.; Chen, J. G.; Pandelov, S.; Stimming, U., Trends in the Exchange Current for Hydrogen Evolution. *J. Electrochem. Soc.* **2005,** *152* (3), J23.

(11) Xie, Z.; Chen, M.; Peera, S. G.; Liu, C.; Yang, H.; Qi, X.; Kumar, U. P.; Liang, T., Theoretical Study on a Nitrogen-Doped Graphene Nanoribbon with Edge Defects as the Electrocatalyst for Oxygen Reduction Reaction. *ACS Omega* **2020,** *5* (10), 5142-5149.

(12) Han, J.; Meng, X.; Lu, L.; Bian, J.; Li, Z.; Sun, C., Single-atom Fe-N_x-C as an efficient electrocatalyst for zinc-air batteries. *Adv. Funct. Mater.* **2019,** *29* (41), 1808872.

(13) Chen, P.; Zhou, T.; Xing, L.; Xu, K.; Tong, Y.; Xie, H.; Zhang, L.; Yan, W.; Chu, W.; Wu, C.; Xie, Y., Atomically dispersed iron-nitrogen species as electrocatalysts for bifunctional oxygen evolution and reduction reactions. *Angew. Chem. Int. Ed.* **2017,** *56* (2), 610-614.

(14) Fei, H.; Dong, J.; Feng, Y.; Allen, C. S.; Wan, C.; Volosskiy, B.; Li, M.; Zhao, Z.; Wang, Y.; Sun, H.; An, P.; Chen, W.; Guo, Z.; Lee, C.; Chen, D.; Shakir, I.; Liu, M.; Hu, T.;

Li, Y.; Kirkland, A. I.; Duan, X.; Huang, Y., General synthesis and definitive structural identification of MN_4C_4 single-atom catalysts with tunable electrocatalytic activities. *Nat. Catal.* **2018,** *1* (1), 63-72.
(15) Chen, J.; Li, H.; Fan, C.; Meng, Q.; Tang, Y.; Qiu, X.; Fu, G.; Ma, T., Dual single-atomic Ni-N_4 and Fe-N_4 sites constructing janus hollow graphene for selective oxygen electrocatalysis. *Adv. Mater.* **2020,** *32* (30), 2003134.
(16) Pan, F.; Jin, T.; Yang, W.; Li, H.; Cao, Y.; Hu, J.; Zhou, X.; Liu, H.; Duan, X., Theory-guided design of atomic Fe-Ni dual sites in N,P-co-doped C for boosting oxygen evolution reaction. *Chem Cata.* **2021,** *1* (3), 734-745.
(17) Yang, G.; Zhu, J.; Yuan, P.; Hu, Y.; Qu, G.; Lu, B.-A.; Xue, X.; Yin, H.; Cheng, W.; Cheng, J.; Xu, W.; Li, J.; Hu, J.; Mu, S.; Zhang, J.-N., Regulating Fe-spin state by atomically dispersed Mn-N in Fe-N-C catalysts with high oxygen reduction activity. *Nat. Commun.* **2021,** *12* (1), 1734.
(18) Wang, X.; Yu, L.; Guan, B. Y.; Song, S.; Lou, X. W., Metal-organic framework hybrid-assisted formation of Co_3O_4/Co-Fe oxide double-shelled nanoboxes for enhanced oxygen evolution. *Adv. Mater.* **2018,** *30* (29), 1801211.
(19) Liu, C.; Qian, J.; Ye, Y.; Zhou, H.; Sun, C.-J.; Sheehan, C.; Zhang, Z.; Wan, G.; Liu, Y.-S.; Guo, J.; Li, S.; Shin, H.; Hwang, S.; Gunnoe, T. B.; Goddard, W. A.; Zhang, S., Oxygen evolution reaction over catalytic single-site Co in a well-defined brookite TiO2 nanorod surface. *Nat. Catal.* **2021,** *4* (1), 36-45.
(20) Zhu, Y.; Tahini, H. A.; Hu, Z.; Chen, Z.-G.; Zhou, W.; Komarek, A. C.; Lin, Q.; Lin, H.-J.; Chen, C.-T.; Zhong, Y.; Fernández-Díaz, M. T.; Smith, S. C.; Wang, H.; Liu, M.; Shao, Z., Boosting oxygen evolution reaction by creating both metal ion and lattice-oxygen active sites in a complex oxide. *Adv. Mater.* **2020,** *32* (1), 1905025.
(21) Guan, B. Y.; Yu, L.; Lou, X. W., General synthesis of multishell mixed-metal oxyphosphide particles with enhanced electrocatalytic activity in the oxygen evolution reaction. *Angew. Chem. Int. Ed.* **2017,** *56* (9), 2386-2389.
(22) Saad, A.; Cheng, Z.; Zhang, X.; Liu, S.; Shen, H.; Thomas, T.; Wang, J.; Yang, M., Ordered mesoporous cobalt–nickel nitride prepared by nanocasting for oxygen evolution reaction electrocatalysis. *Adv. Mater. Interfaces* **2019,** *6* (20), 1900960.
(23) Walter, C.; Menezes, P. W.; Orthmann, S.; Schuch, J.; Connor, P.; Kaiser, B.; Lerch, M.; Driess, M., A molecular approach to manganese nitride acting as a high performance electrocatalyst in the oxygen evolution reaction. *Angew. Chem. Int. Ed.* **2018,** *57* (3), 698-702.

(24) Yuan, Y.; Adimi, S.; Guo, X.; Thomas, T.; Zhu, Y.; Guo, H.; Priyanga, G. S.; Yoo, P.; Wang, J.; Chen, J.; Liao, P.; Attfield, J. P.; Yang, M., A surface-oxide-rich activation layer (SOAL) on Ni_2Mo_3N for a rapid and durable oxygen evolution reaction. *Angew. Chem. Int. Ed.* **2020,** *59* (41), 18036-18041.
(25) Chen, P.; Xu, K.; Fang, Z.; Tong, Y.; Wu, J.; Lu, X.; Peng, X.; Ding, H.; Wu, C.; Xie, Y., Metallic Co_4N porous nanowire arrays activated by surface oxidation as electrocatalysts for the oxygen evolution reaction. *Angew. Chem. Int. Ed.* **2015,** *54* (49), 14710-14714.
(26) Stern, L.-A.; Feng, L.; Song, F.; Hu, X., Ni_2P as a janus catalyst for water splitting: the oxygen evolution activity of Ni_2P nanoparticles. *Energy Environ. Sci.* **2015,** *8* (8), 2347-2351.
(27) He, P.; Yu, X.-Y.; Lou, X. W., Carbon-incorporated nickel-cobalt mixed metal phosphide nanoboxes with enhanced electrocatalytic activity for oxygen evolution. *Angew. Chem. Int. Ed.* **2017,** *56* (14), 3897-3900.
(28) Li, D.; Baydoun, H.; Verani, C. N.; Brock, S. L., Efficient water oxidation using CoMnP nanoparticles. *J. Am. Chem. Soc.* **2016,** *138* (12), 4006-4009.
(29) Hou, Y.; Liu, Y.; Gao, R.; Li, Q.; Guo, H.; Goswami, A.; Zboril, R.; Gawande, M. B.; Zou, X., Ag@Co_xP core-shell heterogeneous nanoparticles as efficient oxygen evolution reaction catalysts. *ACS Catal.* **2017,** *7* (10), 7038-7042.
(30) Yang, S.; Chen, G.; Ricciardulli, A. G.; Zhang, P.; Zhang, Z.; Shi, H.; Ma, J.; Zhang, J.; Blom, P. W. M.; Feng, X., Topochemical synthesis of two-dimensional transition-metal phosphides using phosphorene templates. *Angew. Chem. Int. Ed.* **2020,** *59* (1), 465-470.
(31) Yu, D.; Ma, Y.; Hu, F.; Lin, C.-C.; Li, L.; Chen, H.-Y.; Han, X.; Peng, S., Dual-sites coordination engineering of single atom catalysts for flexible metal-air batteries. *Adv. Energy Mater.* **2021,** *11* (30), 2101242.
(32) Li, S.; Cheng, C.; Zhao, X.; Schmidt, J.; Thomas, A., Active salt/silica-templated 2D mesoporous FeCo-N_x-carbon as bifunctional oxygen electrodes for zinc-air batteries. *Angew. Chem. Int. Ed.* **2018,** *57* (7), 1856-1862.
(33) Xiao, M.; Xing, Z.; Jin, Z.; Liu, C.; Ge, J.; Zhu, J.; Wang, Y.; Zhao, X.; Chen, Z., Preferentially engineering FeN_4 edge sites onto graphitic nanosheets for highly active and durable oxygen electrocatalysis in rechargeable zn-air batteries. *Adv. Mater.* **2020,** *32* (49), 2004900.
(34) Guo, Y.; Yuan, P.; Zhang, J.; Hu, Y.; Amiinu, I. S.; Wang, X.; Zhou, J.; Xia, H.; Song, Z.; Xu, Q.; Mu, S., Carbon nanosheets containing discrete Co-N_x-B_y-C active sites for efficient oxygen electrocatalysis and rechargeable zn-air batteries. *ACS Nano* **2018,** *12* (2), 1894-1901.

(35) Yang, H. B.; Miao, J.; Hung, S.-F.; Chen, J.; Tao, H. B.; Wang, X.; Zhang, L.; Chen, R.; Gao, J.; Chen, H. M.; Dai, L.; Liu, B., Identification of catalytic sites for oxygen reduction and oxygen evolution in N-doped graphene materials: Development of highly efficient metal-free bifunctional electrocatalyst. *Sci. Adv.* **2016,** *2* (4), e1501122.
(36) Sun, T.; Wang, J.; Qiu, C.; Ling, X.; Tian, B.; Chen, W.; Su, C., B, N codoped and defect-rich nanocarbon material as a metal-free bifunctional electrocatalyst for oxygen reduction and evolution reactions. *Adv. Sci.* **2018,** *5* (7), 1800036.
(37) Chen, S.; Zhao, L.; Ma, J.; Wang, Y.; Dai, L.; Zhang, J., Edge-doping modulation of N, P-codoped porous carbon spheres for high-performance rechargeable Zn-air batteries. *Nano Energy* **2019,** *60*, 536-544.
(38) Lei, W.; Deng, Y.-P.; Li, G.; Cano, Z. P.; Wang, X.; Luo, D.; Liu, Y.; Wang, D.; Chen, Z., Two-dimensional phosphorus-doped carbon nanosheets with tunable porosity for oxygen reactions in zinc-air batteries. *ACS Catal.* **2018,** *8* (3), 2464-2472.
(39) Tam, T. V.; Kang, S. G.; Kim, M. H.; Lee, S. G.; Hur, S. H.; Chung, J. S.; Choi, W. M., Novel graphene hydrogel/B-doped graphene quantum dots composites as trifunctional electrocatalysts for zn-air batteries and overall water splitting. *Adv. Energy Mater.* **2019,** *9* (26), 1900945.
(40) Wang, Q.; Lei, Y.; Zhu, Y.; Wang, H.; Feng, J.; Ma, G.; Wang, Y.; Li, Y.; Nan, B.; Feng, Q.; Lu, Z.; Yu, H., Edge defect engineering of nitrogen-doped carbon for oxygen electrocatalysts in an-air batteries. *ACS Appl. Mater. Inter.* **2018,** *10* (35), 29448-29456.
(41) Ji, D.; Fan, L.; Tao, L.; Sun, Y.; Li, M.; Yang, G.; Tran, T. Q.; Ramakrishna, S.; Guo, S., The Kirkendall effect for engineering oxygen vacancy of hollow Co_3O_4 nanoparticles toward high-performance portable zinc-air batteries. *Angew. Chem. Int. Ed.* **2019,** *58* (39), 13840-13844.
(42) Han, X.; He, G.; He, Y.; Zhang, J.; Zheng, X.; Li, L.; Zhong, C.; Hu, W.; Deng, Y.; Ma, T.-Y., Engineering catalytic active sites on cobalt oxide surface for enhanced oxygen electrocatalysis. *Adv. Energy Mater.* **2018,** *8* (10), 1702222.
(43) Aijaz, A.; Masa, J.; Rösler, C.; Xia, W.; Weide, P.; Botz, A. J. R.; Fischer, R. A.; Schuhmann, W.; Muhler, M., Co@Co_3O_4 encapsulated in carbon nanotube-grafted nitrogen-doped carbon polyhedra as an advanced bifunctional oxygen electrode. *Angew. Chem. Int. Ed.* **2016,** *55* (12), 4087-4091.
(44) Wang, X.-T.; Ouyang, T.; Wang, L.; Zhong, J.-H.; Ma, T.; Liu, Z.-Q., Redox-inert Fe^{3+} ions in octahedral sites of Co-Fe spinel oxides with enhanced oxygen catalytic activity for rechargeable zinc-air batteries. *Angew. Chem. Int. Ed.* **2019,** *58* (38), 13291-13296.

(45) Liu, Z.-Q.; Cheng, H.; Li, N.; Ma, T. Y.; Su, Y.-Z., $ZnCo_2O_4$ quantum dots anchored on nitrogen-doped carbon nanotubes as reversible oxygen reduction/evolution electrocatalysts. *Adv. Mater.* **2016,** *28* (19), 3777-3784.
(46) Wang, Q.; Shang, L.; Shi, R.; Zhang, X.; Waterhouse, G. I. N.; Wu, L.-Z.; Tung, C.-H.; Zhang, T., 3D carbon nanoframe scaffold-immobilized Ni_3FeN nanoparticle electrocatalysts for rechargeable zinc-air batteries' cathodes. *Nano Energy* **2017,** *40*, 382-389.
(47) Cui, Z.; Fu, G.; Li, Y.; Goodenough, J. B., Ni_3FeN-supported Fe_3Pt intermetallic nanoalloy as a high-performance bifunctional catalyst for metal-air batteries. *Angew. Chem. Int. Ed.* **2017,** *56* (33), 9901-9905.
(48) Zhu, C.; Yin, Z.; Lai, W.; Sun, Y.; Liu, L.; Zhang, X.; Chen, Y.; Chou, S.-L., Fe-Ni-Mo nitride porous nanotubes for full water splitting and zn-air batteries. *Adv. Energy Mater.* **2018,** *8* (36), 1802327.
(49) Zheng, X.; Han, X.; Cao, Y.; Zhang, Y.; Nordlund, D.; Wang, J.; Chou, S.; Liu, H.; Li, L.; Zhong, C.; Deng, Y.; Hu, W., Identifying dense $NiSe_2$/$CoSe_2$ heterointerfaces coupled with surface high-valence bimetallic sites for synergistically enhanced oxygen electrocatalysis. *Adv. Mater.* **2020,** *32* (26), 2000607.
(50) Zheng, X.; Zhang, J.; Wang, J.; Zhang, Z.; Hu, W.; Han, Y., Facile synthesis of nickel cobalt selenide hollow nanospheres as efficient bifunctional electrocatalyst for rechargeable Zn-air battery. *Science China Materials* **2020,** *63* (3), 347-355.
(51) Zeng, Z.; Fu, G.; Yang, H. B.; Yan, Y.; Chen, J.; Yu, Z.; Gao, J.; Gan, L. Y.; Liu, B.; Chen, P., Bifunctional N-$CoSe_2$/3D-MXene as highly efficient and durable cathode for rechargeable zn-air battery. *ACS Materials Lett.* **2019,** *1* (4), 432-439.
(52) Park, J.; Risch, M.; Nam, G.; Park, M.; Shin, T. J.; Park, S.; Kim, M. G.; Shao-Horn, Y.; Cho, J., Single crystalline pyrochlore nanoparticles with metallic conduction as efficient bi-functional oxygen electrocatalysts for Zn–air batteries. *Energy Environ. Sci.* **2017,** *10* (1), 129-136.
(53) Bian, J.; Su, R.; Yao, Y.; Wang, J.; Zhou, J.; Li, F.; Wang, Z. L.; Sun, C., Mg doped perovskite $LaNiO_3$ nanofibers as an efficient bifunctional catalyst for rechargeable zinc-air batteries. *ACS Appl. Energy Mater.* **2019,** *2* (1), 923-931.
(54) Ran, J.; Wang, T.; Zhang, J.; Liu, Y.; Xu, C.; Xi, S.; Gao, D., Modulation of electronics of oxide perovskites by sulfur doping for electrocatalysis in rechargeable zn-air batteries. *Chem. Mater.* **2020,** *32* (8), 3439-3446.

(55) Wang, X.; Sunarso, J.; Lu, Q.; Zhou, Z.; Dai, J.; Guan, D.; Zhou, W.; Shao, Z., High-performance platinum-perovskite composite bifunctional oxygen electrocatalyst for rechargeable zn-air battery. *Adv. Energy Mater.* **2020,** *10* (5), 1903271.
(56) Li, Z.; Niu, W.; Yang, Z.; Zaman, N.; Samarakoon, W.; Wang, M.; Kara, A.; Lucero, M.; Vyas, M. V.; Cao, H.; Zhou, H.; Sterbinsky, G. E.; Feng, Z.; Du, Y.; Yang, Y., Stabilizing atomic Pt with trapped interstitial F in alloyed PtCo nanosheets for high-performance zinc-air batteries. *Energy Environ. Sci.* **2020,** *13* (3), 884-895.
(57) Luo, M.; Zhao, Z.; Zhang, Y.; Sun, Y.; Xing, Y.; Lv, F.; Yang, Y.; Zhang, X.; Hwang, S.; Qin, Y.; Ma, J.-Y.; Lin, F.; Su, D.; Lu, G.; Guo, S., PdMo bimetallene for oxygen reduction catalysis. *Nature* **2019,** *574* (7776), 81-85.
(58) Pan, F.; Li, Z.; Yang, Z.; Ma, Q.; Wang, M.; Wang, H.; Olszta, M.; Wang, G.; Feng, Z.; Du, Y.; Yang, Y., Porous FeCo glassy alloy as bifunctional support for high-performance zn-air battery. *Adv. Energy Mater.* **2021,** *11* (3), 2002204.
